# Revealing the mechanism of raw and vinegar-processed *Curcuma aromatica* Salisb. [Zingiberaceae] regulates primary dysmenorrhea in rats *via* integrated metabolomics

**DOI:** 10.3389/fphar.2022.926291

**Published:** 2022-09-13

**Authors:** Lianlin Su, Huangjin Tong, Jiuba Zhang, Min Hao, Chenghao Fei, De Ji, Wei Gu, Zhenhua Bian, Chunqin Mao, Tulin Lu

**Affiliations:** ^1^ College of Pharmacy, Nanjing University of Chinese Medicine, Nanjing, China; ^2^ Affiliated Hospital of Integrated Traditional Chinese and Western Medicine, Nanjing University of Chinese Medicine, Nanjing, China; ^3^ Jiangsu Province Academy of Traditional Chinese Medicine, Nanjing, China; ^4^ College of Pharmacy, Zhejiang Chinese Medical University, Hangzhou, China; ^5^ Wuxi TCM Hospital Affiliated to Nanjing University of Chinese Medicine, Wuxi, China

**Keywords:** *Curcuma aromatica* Salisb. [Zingiberaceae], vinegar processing, primary dysmenorrhea, metabolomics, multivariate statistical analysis

## Abstract

Primary dysmenorrhea (PDM) is a common disorder among women around the world. Two processed products of *Curcuma aromatica* Salisb. [Zingiberaceae] (CAS) are traditional Chinese medicine (TCM) that have long been used to treat gynecological blood stasis syndrome such as primary dysmenorrhea. The mechanisms and active substances of CAS are still largely unknown. The study aimed to establish a rat model of primary dysmenorrhea which investigates the differences between the pharmacodynamics and mechanisms of raw CAS (RCAS) and vinegar-processed CAS (VCAS). Histopathology, cytokinetics, and metabolomics were adopted to evaluate the anti-blood stasis effect of RCAS and VCAS. In metabolomics, endogenous differential metabolites in plasma, urine, and feces are the essential steps to evaluate the effect of RCAS and VCAS. In this study, the rat model of primary dysmenorrhea was successfully established. After RCAS and VCAS intervention, the uterine tissue morphology of dysmenorrhea model rats was improved, and gland hypertrophy and myometrial hyperplasia were reduced as well as neutrophil content. Compared with the RCAS group, the VCAS group had better uterine morphology, few inflammatory factors, and significantly improved amino acid and lipid metabolism. The aforementioned results support the conclusion that VCAS performed better than RCAS in primary dysmenorrhea and that vinegar processing increases the efficacy of CAS.

## 1 Introduction

Primary dysmenorrhea (PDM) refers to the pain and distention of the lower abdomen before, during, and/or after menstruation caused by non-organic lesions of the reproductive organs. PDM is often accompanied by low back pain or other discomforts, all belonging to the category of “menstrual abdominal pain” (Chai et al., 2020). Epidemiological studies show that primary dysmenorrhea is the most common disease in gynecology, affecting 25–95% of women around the world, with 10% having serious symptoms. This is one of the most common and frequent causes for disruptions in women’s normal work and deterioration of their quality of life (De Sanctis et al., 2015; Nguyen et al., 2015; Sahin et al., 2018). According to TCM, dysmenorrhea is mainly caused by “pain when *Qi* and *Blood* are blocked” or “pain when the uterus is not well-nourished.” It is also often caused by damp heat accumulation, kidney qi deficiency, cold coagulation and blood stasis, qi stagnation and blood stasis, and *Qi* and *Blood* weakness.

According to modern research, the occurrence of primary dysmenorrhea is mainly related to increased proportion of prostaglandins (PGs), mainly PGF2 *a*/PGE2, in the endometrium during menstruation (Chan 1983; Maia et al., 2005). PGF2 *a* promotes contraction of the uterine smooth muscle and vasospasm, which can cause pain due to local ischemia and hypoxia, while PGE2 has the opposite reaction (Ju et al., 2014; Iacovides et al., 2015). In addition, related studies have shown that primary dysmenorrhea is also related to tumor necrosis factor (TNF - α), interleukin (IL), plasma thromboxane B2 (TXB2), 6-keto-PGF1 α, NO, Ca^2+^, and beta-endorphin (β - EP) (Dawood and Khan-Dawood, 2007a; Pu et al., 2014; Xie et al., 2014; Huang et al., 2016). At present, the treatment of primary dysmenorrhea in Western medicine is not yet satisfactory: non-steroidal anti-inflammatory drugs and oral contraceptives have limited clinical use due to their serious adverse reactions (Oladosu et al., 2018). TCM has many effective methods in the treatment of primary dysmenorrhea and has unique advantages in the improvement of dysmenorrhea symptoms and long-term curative effects (Xu et al., 2019). In the treatment of primary dysmenorrhea, ancient and modern doctors started with blood stasis, which is the main syndrome type. The main treatment methods revolve around promoting blood circulation and removing blood stasis. TCM treatments for promoting blood circulation and removing blood stasis can improve microcirculation, dilate blood vessels, and change hemorheology, all of which are significant and effective ways to relieve pain.

The dried rhizomes of CAS, a ginger plant, are spicy, bitter, and warm in nature. It promotes *Qi* and breaking blood, eliminating accumulation and relieving pain. It is used for treating syndromes such as lump, blood stasis and amenorrhea, chest arthralgia and heartache, food accumulation, and flatulence. According to the 2020 edition of Chinese Pharmacopoeia (Chinese Pharmacopoeia, 2020), through different processing methods of steaming and vinegar, two processed products of CAS, RCAS, and VCAS, were prepared. Both RCAS and VCAS have the effect of removing blood stasis and pain, but their clinical applications have differences despite their similarity (Liu et al., 2017; Xie et al., 2020). CAS can relieve pain by promoting *Qi*, breaking blood, and removing blood stasis, and these effects are enhanced after vinegar processing. VCAS has a very high frequency in the clinical application of primary dysmenorrhea. For example, there are 18 prescriptions containing VCAS in the Chinese Pharmacopoeia 2020 edition, which mainly treat blood stasis-related diseases (Hao et al., 2018; Chinese Pharmacopoeia, 2020). In addition, *Fufang Ezhusan* uses VCAS as the principal medicine for treating dysmenorrhea of blood stasis type. Moreover, clinical and animal studies of formulae containing VCAS in the treatment of primary dysmenorrhea are also more common than those of RCAS (Gu et al., 2018; Hao et al., 2018). However, few reports can be found on the mechanism of enhancing stasis and relieving pain and relieving dysmenorrhea after vinegar processing. As a result, VCAS can enhance the treatment of primary dysmenorrhea which deserves further study.

Ultra-performance liquid chromatography–quadrupole–time-of-flight mass spectrometry (UHPLC-Q/TOF-MS) has the characteristics of high resolution, sensitivity, simplicity, and high-throughput. It has been used to successfully study metabolic changes caused by diseases and drug toxicity (Su et al., 2019). In this study, metabolomics technology based on UHPLC-Q/TOF-MS and multivariate statistics were used to screen out potential biomarkers in blood, urine, and fecal metabolites related to primary dysmenorrhea, analyze related metabolic pathways, and compare the efficacy and mechanism of RCAS and VCAS in the treatment of primary dysmenorrhea.

This study provides a basis for further research on the mechanism of VCAS in the treatment of primary dysmenorrhea. Through the in-depth analysis of dysmenorrhea-related pain factors, related metabolites, and metabolic pathways, it is found that metabolomics can comprehensively demonstrate the impact of the disease on the whole body, and the selected indicators can holistically reflect the state of the disease and provide a new approach for the study of mechanism, screening, or clinical drug treatment of diseases related to blood stasis syndrome.

## 2 Materials and methods

### 2.1 Preparation of RCAS and VCAS extracts

The fresh rhizome of CAS was purchased from Hebei Anguo juyaotang Pharmaceutical Co., Ltd. (#1810003), Hebei, China in December 2018. The samples were identified as the rhizome of *Curcuma aromatica* Salisb. [Zingiberaceae] by Professor Jianwei Cheng at Nanjing University of Chinese Medicine.

RCAS slices: RCAS was steamed for 2 h, cut into 3-mm thickness, and dried in the oven.

VCAS slices: RCAS was boiled with vinegar (per 100 kg samples with 20 kg vinegar and percentile of vinegar is 20% approximately), and dried in the oven at 40°C (Chinese Pharmacopoeia, 2020).

The RCAS and VCAS slices were refluxed for 1.5 h with 90% ethanol 15 times and extracted twice. The residues were extracted for 1.5 h with water 10 times. The aforementioned extracts of RCAS and VCAS were combined. After filtrating and concentrating, per 1 ml RCAS and VCAS extracts containing 1 g of raw medicines were prepared (Xie et al., 2020).

### 2.2 Chemicals and reagents

LC-MS-grade acetonitrile, LC-MS-grade methyl alcohol, HPLC-grade methanoic acid (Merck. Co. Inc., Darmstadt, Germany), and ultra-pure-grade water were obtained from a Milli-Q system (Millipore, Bedford, MA, United States). The other solvents were of analytical grade.

Epinephrine hydrochloride injection (#10180505) was purchased from Harvest Pharmaceutical Co., Ltd. (Shanghai, China). Estradiol benzoate (#20190301) injection was purchased from Jinke Pharmaceutical Co., Ltd. (Sichuan, China). Oxytocin injection (#190207) was purchased from Hongye Pharmaceutical Co., Ltd. (Anhui, China). *Tong Jing Bao Ke Li* (#181203) was purchased from Zhongjingwanxi Pharmaceutical Co., Ltd. (Henan, China). The ELISA kits of interleukin-6 (IL-6, #EK306-01), tumor necrosis factor-*a* (TNF-α, #EK382HS-01), 6-keto-prostaglandin F1a (6-keto-PGF1a, #E-EL-0054c), prostaglandin E2 (PGE2, #E-EL-0034c), prostaglandin F2α (PG F2α, #E-EL-R0795c), thromboxane B2 (TXB2, #E-EL-R0965c), and beta-endorphin (β-EP, #E-EL-R0105c) were purchased from Nanjing Kaiji Technology Co., Ltd., Nanjing, China. The colorimetric assay kits of calcium (Ca^2+^, #C004-2-1) and nitric oxide (NO, #A013-2-1) were provided by Jiangcheng Bioengineering Institute, Nanjing, China.

### 2.3 Chemical analysis of RCAS and VCAS extracts

RCAS and VCAS extracts were prepared with a reverse phase-solid phase extract column (RP-SPE) by gradient elution, and the analysis determination performance by UHPLC-Q/TOF-MS coupled with chemometric analysis according to literature and compared the differences in the chemical profiles of RCAS and VCAS (Hao et al., 2018). At the same time, the volatile oil content in RCAS and VCAS was analyzed by GC-MS. However, due to the thermal instability of very few sesquiterpenoids, this part of the research was just a supplement to UHPLC-Q/TOF-MS (Yang et al., 2007). The results of the chemical composition analysis are detailed in the Supplementary Materials.

### 2.4 Animals and drug administration

A total of 70 specific pathogen-free (SPF) degree female Sprague–Dawley (SD) rats (180 ± 20 g) were obtained from the animal breeding farm of Qinglong Mountain in Jiangning District, Nanjing, China (license approval number: SCXK (SU) 2019-0001). Before the experiment, the rats were fed in an environment-controlled breeding room. A 12-h light/dark cycle was set. Room temperature and relative humidity were regulated at 25 ± 2°C and 60 ± 5%, respectively. In this study, all experimental animals were allowed free access to food and tap water. All rats were adapted for 7 days before the experiment. The study protocol was in accordance with the Guide for the Care and Use of Laboratory Animals and was approved by the Animal Experimental Ethics Committee of the Nanjing University of Chinese Medicine.

A total of 40 rats were randomly divided into five groups with each group having eight rats. Five groups were set as below: the normal control group (NC), model group (M), RCAS, VCAS, and *Tong Jingbao* group (TJB, a Chinese patent medicine granule), which was the positive control group. TJB, RCAS, and VCAS were intragastrically administered to the TJB group (2.1 g/kg/day), RCAS group (3.8 g/kg/day), and VCAS group (3.8 g/kg/day), respectively, for the last 7 days. The dose of administration was changed according to the clinical equivalent dose of rats. NC and M groups were intragastrically administered normal saline. The models in M, TJB, RCAS, and VCAS groups were established on the 10th day after the dose according to the references: subcutaneous injected 0.1% adrenaline (0.9 mg/kg/d) for 10 days, and 4 h later, the rats were given comprehensive stimulation [A: sound stimulation (60 dB, (10 ± 5) Hz] for 10 min; B: light stimulation [(2 ± 1) Hz) for 10 min; C: binding for 10 min; D: nipping tail for 10 min; E: swimming in ice water (0–4°C) for 5 min]. The models were subcutaneously injected with 0.2% estradiol benzoate for 10 days, given a dose (2.5 mg/kg/d) on the 1st and 10th day, and another dose (1.25 mg/kg/d) from the 2nd to the 9th day. After the last estradiol benzoate injection, 1 h later, the rats were intraperitoneally injected with oxytocin (10 U/kg), while NC groups were intragastrically administered normal saline. After that, all rats were intraperitoneally injected with 1% pentobarbital sodium (50 mg/kg, i.p). Blood samples were collected by the abdominal aortic method.

### 2.5 Sample collection

The experiment continued for 10 days. At the end of treatment, the rats were euthanized, and blood samples were collected by heparin sodium blood collection tubes. The samples were centrifuged at 3000 r/min for 10 min, and then the supernatants were stored at −80°C for related function detection. Urine and feces samples were collected in the 12 h after drug administration and stored at −80°C for UHPLC-Q/TOF-MS analysis.

### 2.6 Sample preparation

Before mass spectrometric detection, the urine and feces samples were unfrozen at ambient temperature. A total of 100-mg feces samples were added to 70% chromatographic pure methanol (2 ml). Eight times the volume of methanol was added to the 200-µl plasma or urine sample. The mixed samples were shaken violently for 30 s by a vortex mixer. Then, the mixture was centrifuged at 12,000 r/min at 4°C for 10 min. The supernate was prepared for mass detection.

### 2.7 Histopathology and writhing response

The uterus of rats was fixed in 10% formalin solution and processed routinely for paraffin embedding. Sections (5 μm thick) were deparaffinized and then stained with hematoxylin and eosin solutions (H&E). Then, the histopathology samples were examined under light microscopy (Leica, DFC-259, and Germany). According to the degree of endometrial hyperplasia, the morphological changes of all rats’ uterus were graded as 0∼ 4: 0 as no pathological phenomenon and 4 as the most serious pathological damage. The specific scoring criteria are as follows: 0: the uterine structure is clear, with a three-layer structure, including the endometrium, myometrium, and adventitia from inside to outside. The endometrial epithelial cells are complete, the distribution of glands in the lamina propria is normal, and the thickness of the endometrium and myometrium is normal. Four points: the uterine structure is disordered, the uterine cavity is not smooth, gland distribution in lamina propria is abnormal, gland hypertrophy, endometrial hyperplasia is obvious, myometrial hyperplasia is obvious, and inflammatory cell infiltration is obvious (Thaina et al., 2009).

On the 10th day of the experiment, after oxytocin was injected into the abdominal cavity of rats in each group, the writhing times and writhing latency of rats within 0–30 min were immediately observed: abdominal concave contraction, trunk and hind limb extension, and hip and one side limb internal rotation. Writhing latency: the time from the beginning of the intraperitoneal injection of oxytocin to the first writhing reaction in rats.

### 2.8 Biochemical assays

The rat uterine sample was washed with normal saline, dried, and weighed. Then, 10% of the tissue homogenate was prepared with pre-cooled normal saline at a ratio of 1:9 (weight: volume). The homogenate was centrifuged at 3000 rpm for 5 min, and the supernatant was stored at −80°C for HYP detection, which was determined by ELISA or colorimetric assay method. PGF2α, PGE2, β-EP, TXB2, 6-keto-PGF1α, TNF-α, IL-6, NO, and Ca^2+^ were carried out in perfect accordance with the product’s instructions (Hao et al., 2018).

### 2.9 Mass spectrum analysis and verification of methodology

The plasma, urine, and feces sample analysis was managed by Shimadzu UPLC (Kyoto, Japan) which consisted of an LC-30AD binary liquid pump, SIL-30SD auto sampler and DGU-20A5R on-line solvent degasser, a CTO-30 A column oven, an AB SCIEX Triple TOF 5600^+^ system, and an ESI source (Hao et al., 2018). Chromatographic conditions were as follows: Agilent C_18_ reversed phase column (2.1 mm × 100 mm, 1.8 µm, Palo Alto, CA, United States), mobile phase A (0.1% formic acid aqueous solution)-B (acetonitrile), gradient elution program: 0–1 min, 5–25% B; 1–3 min, 25–30% B; 3–13 min, 30–55% B; 13–15 min, 55–70% B; 15–25 min, 70–100% B; 25–28 min, 5–100% B; flow rate: 0.3 ml/min; column temperature: 35°C; injection volume: 1 µl. Mass spectrometer condition: ESI source and data collection in positive and negative ion mode. The source parameters were set as follows: ion spray voltage floating: +4500/− 4500; decluttering potential: +60/− 60 V; source temperature: 550°C; the atomizing gas is N_2_, curtain gas: 35 psi; gas1 (nebulizer gas): 55 psi; gas 2 (heater gas): 55 psi; collision energy: +35/− 35e V; using MS/MS secondary mass spectrometry mode: the MS spectrometer ion scanning range was *m/z* 100–2000. The MS/MS spectrometer ion scanning range was *m/z* 50–1,000, and dynamic background subtraction was turned on. The quality control (QC) sample was obtained by even mixing of all groups of each kind of sample to ensure system suitability. Before the samples started testing, the QC sample was continuously detected six times. Moreover, system consistency was verified by QC samples after every five detected samples.

### 2.10 Multivariate statistical analysis

Analyst TF 1.6 software (AB Sciex, Boston, MA, United States) was used to extract the original metabolic fingerprint profiles. MarkerView1.2.1 (AB Sciex, United States) software was used for peak detection and calibration of the original samples of each group. The parameters were set as follows: retention time range: 0.5∼30 min; quality range: 100–1,200 Da; retention time error limit: 0.01 min; mass error limit: 10 ppm; peak intensity threshold: 10; minimum peak width: 25 ppm; noise threshold: 100; retention time error range: 0.5 min. After the original map of each sample was calibrated and normalized by software, they were converted into a three-dimensional matrix containing the peak name (Rt-*m/z*) sample number and normalized peak area. The three-dimensional matrices of each group were imported into SIMCA-P 14.1 software (Umetrics AB, Sweden). The data were pretreated with Pareto-scaling, followed by PCA and OPLS-DA multivariate statistical analysis. The model predictors are R^2^X (cum) and Q^2^X (cum), with R^2^X (cum) representing the ability of the variable to interpret the model and Q^2^X (cum) signifying the ability to predict the model. The applicability of the model was good when R^2^X (cum) and Q^2^X (cum) both approached 1.0. In the OPLS-DA model, the ions with VIP values greater than 1.0 were identified as potential differential ions. At the same time, *t*-test single-dimensional statistical analysis was carried out on the three-dimensional matrices among different groups. *p* < 0.05 was determined as the ion with a significant difference. Potential differential ions with VIP >1.0 and *p* < 0.05 were confirmed as the final differential ions after taking the intersection, whose structures were to be identified later (Yao et al., 2014).

### 2.11 Biomarker identification and metabolic pathway analysis

The online database One-Map (http://www.5omics.com/) was used for identification of the differential metabolites of plasma, urine, and feces. Excel data (including sample ID, mass/charge ratio/retention time, and ion strength) of all samples after the original chromatogram dimensionality reduction were imported into the One-MAP online cloud platform, and the differential metabolite search and metabolic pathway analysis were automatically carried out. According to the variable importance for the project (VIP) of the OPLS-DA model in each group, the differential ions were selected. Referring to the literature on metabolomics, this study set VIP > 1 as the differential ions between the two groups with independent sample *t*-test, excluding data with *p* > 0.05 (Xing et al., 2017; Su et al., 2019). Finally, the *p* < 0.05 differential ions were input into the HMDB database, the structure of the compounds according to the first-order mass spectrometry information was predicted, the predicted exogenous compounds were eliminated, and the final selected differential compounds for structure identification were obtained, which are compared with the HMDB database (http://www.hmdb.ca/), METLIN database (https://www.sisweb.com/software/ms/wiley-metlin.htm), and KEGG database (http://www.genome.jp/kegg/). The standard secondary mass spectrometry data and related literature were compared, and finally the endogenous differential metabolites were identified and the related metabolic pathways were analyzed.

## 3 Results and discussion

### 3.1 Histopathological and writhing response examination

**FIGURE 1:**
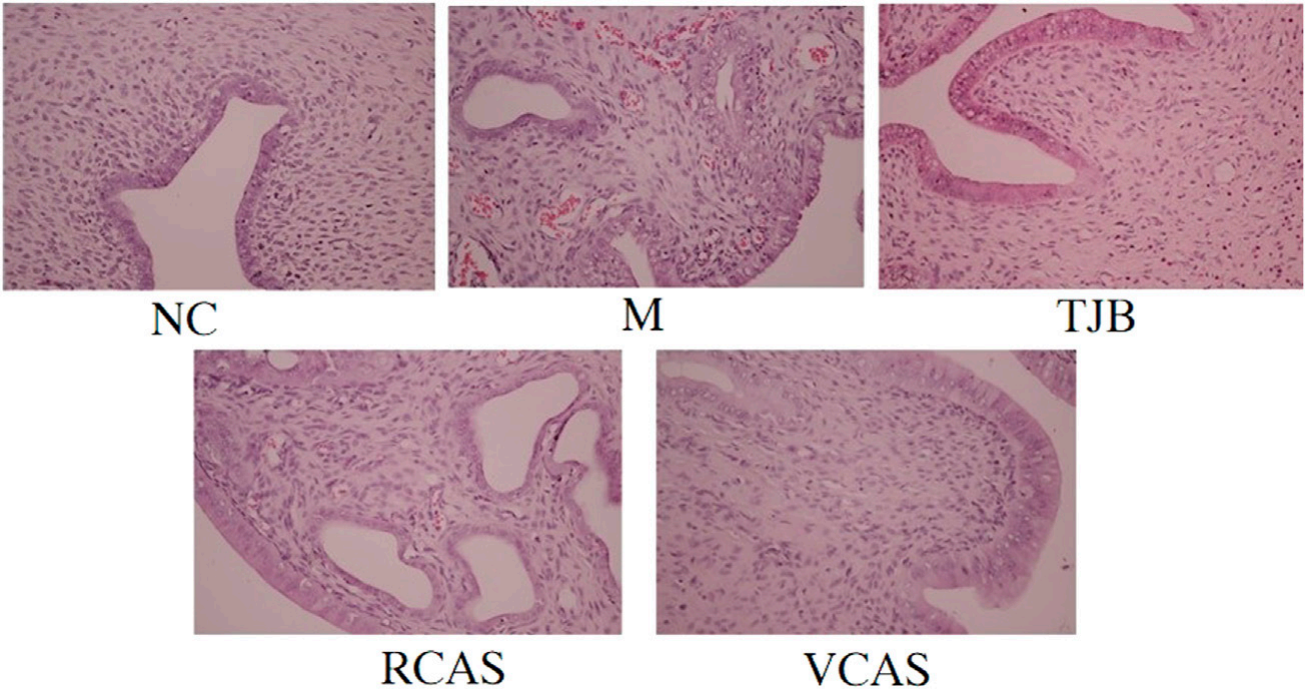
Histological morphology examinations of the uterus (× 400). NC: normal control group, M: model group, TJB: positive drug group, RCAS: raw *Curcuma aromatica* Salisb. [Zingiberaceae], VCAS: vinegar processed *Curcuma aromatica* Salisb. [Zingiberaceae].

The results are shown in Figure 1 and Table 1. As demonstrated in the NC group, the uterine tissue structure of rats in the blank control group was clear, including the endometrium, myometrium, and adventitia from inside to outside, arranged neatly and closely. Of note, the uterine wall became thinner, the structure was disordered, the endometrium was incomplete, the gland was hypertrophied, and the myometrium was obviously proliferated in the M group. There were a lot of inflammatory cells infiltrated, among which neutrophils were the main cells, and the pathological score had a significant difference (*p* < 0.01). In the TJB group, the uterine structure was clear, the endometrium was complete, and the myometrium hyperplasia was not obvious. There was a small amount of neutrophil infiltration, and the pathological score was significantly different (*p* < 0.01). Compared with the M group, the other groups had more regular uterine tissue morphology, less glandular hypertrophy and myometrial hyperplasia, and less neutrophil content (*p* < 0.05 and *p* < 0.01), especially the VCAS group. Moreover, there was no significant improvement in the dysmenorrhea uterus in the RCAS group (*p* > 0.05).

**TABLE 1:** Writhing response of each group during the experiment ( $\bar{x}$ ± s, *n* = 8).

Group	Dosage (g/kg)	Incubation (min)	Numbers of torsion spasm 30 min
NC	—	—	0
M	—	5.19 ± 1.08^##^	10.88 ± 2.53^##^
TJB	2.10	7.49 ± 1.15**	6.63 ± 1.60**
RCAS	3.80	6.43 ± 1.45	9.50 ± 3.16
VCAS	3.80	7.24 ± 1.06**	7.25 ± 1.49**

Compared with the NC group, #*p* ＜ 0.0 and ##*p* ＜ 0.01; compared with the M group, *p ＜ 0.05 and **p ＜ 0.01.

Compared with the model group, the TJB group can significantly prolong the latency of writhing and reduce the number of writhing in the primary dysmenorrhea model rats with qi stagnation and blood stasis (*p* < 0.01); the RCAS group had no statistical significance in writhing latency and writhing times; the VCAS group can significantly prolong the incubation period of writhing and reduce the number of writhing in rats with primary dysmenorrhea caused by qi stagnation and blood stasis (*p* < 0.05 and *p* < 0.01), that is, the effect of the VCAS group is significantly better than that of the RCAS group. The results are shown in Table 1.

### 3.2 Cytokines examinations by ELISA

**FIGURE 2:**
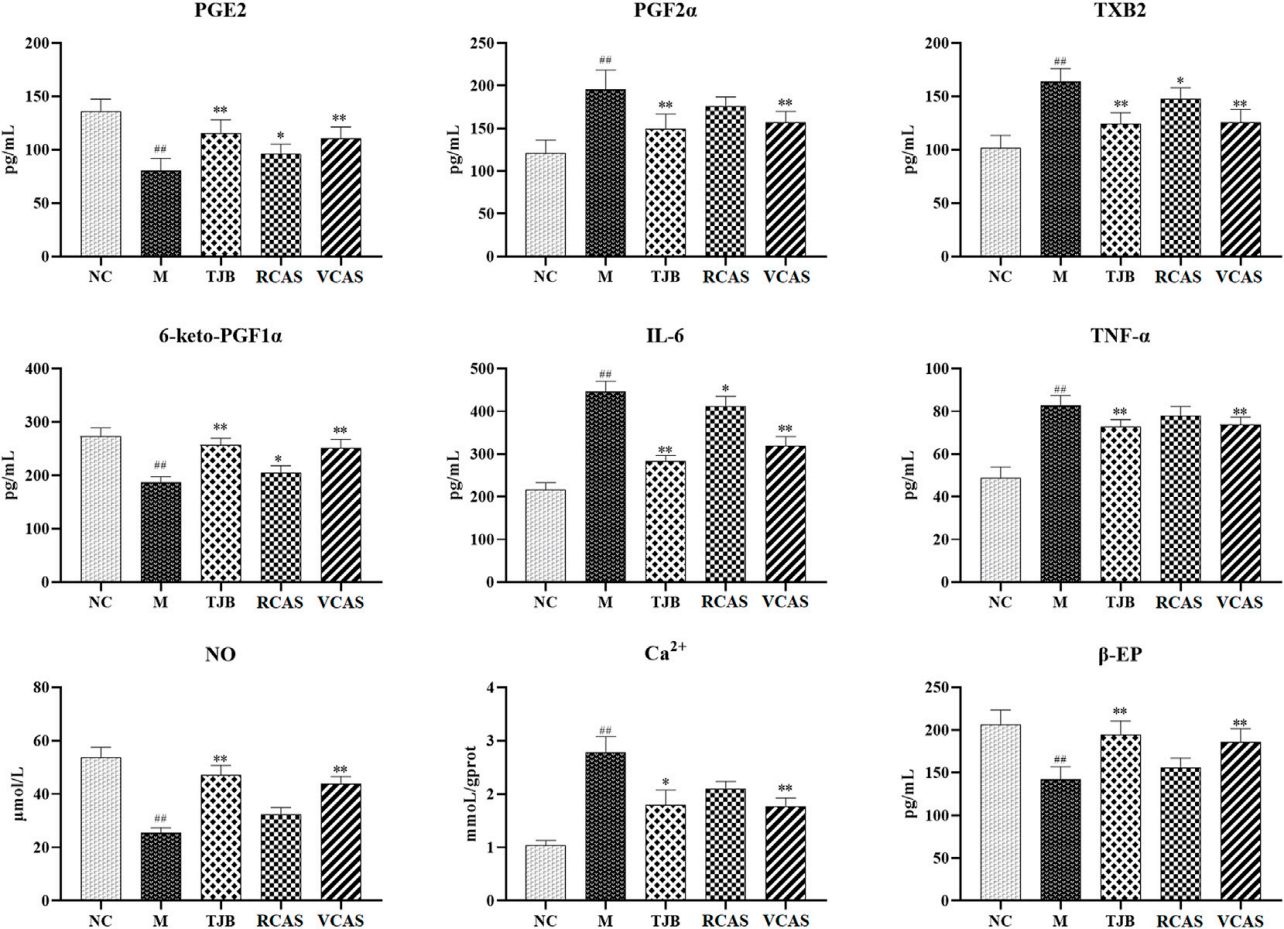
Effects of RCAS and VCAS on pain factors in rats with primary dysmenorrhea. ^##^ and ^#^ represent the comparison with the NC group: *p* < 0.01, *p* < 0.05; ** and * represent the comparison with the M group: *p* < 0.01 and *p* < 0.05.

The results are shown in Figure 2; compared with the NC group, PGE2 and 6-Keto-PGF1α (PG/ml) in the M group decreased significantly (*p* < 0.01), PGF2α and TXB2 increased significantly (*p* < 0.01), and PGE2 and 6-Keto-PGF1α(PG/mL) in the other administration groups were increased when compared with the M group, but PGF2α and TXB2 decreased. Moreover, the most significant change was found in TJB and VCAS (*p* < 0.01). For the inflammatory factors TNF-α and IL-6, the M group increased significantly (*p* < 0.01) but decreased significantly after administration, and the anti-inflammatory effect in TJB and VCAS was significantly increased (*p* < 0.01). Although TNF-α and IL-6 decreased in RCAS, they were not evident and had no statistical significance. Compared with the NC group, the content of Ca ^2+^ in the uterus tissue of the M group was significantly increased, and the content of NO was significantly decreased (*p* < 0.01). The Ca^2+^ in the administration group was decreased and NO was increased, especially in TJB and VCAS (*p* < 0.01). In the M group, β - EP significantly decreased, which plays an important role in the regulation of blood vessels. The decrease of β - EP concentration and activity will lead to vasoconstriction. The administration group can reduce the content of β - EP in brain tissue and increase the content of β - EP in plasma so as to inhibit vasoconstriction, while all groups of administration have an upward trend, in which the increase of TJB and VCAS is statistically significant. The VCAS group significantly increased the contents of PGE2, 6-Keto-PGF1a, and NO and significantly reduced TXB2, IL-6, and TNF- *a* content (*p* < 0.05). Of note, the VCAS group has a more significant role in regulating pain-related factors than the RCAS group.

### 3.3 Plasma metabolomics analysis

#### 3.3.1 Multivariate statistical analysis

**FIGURE 3:**
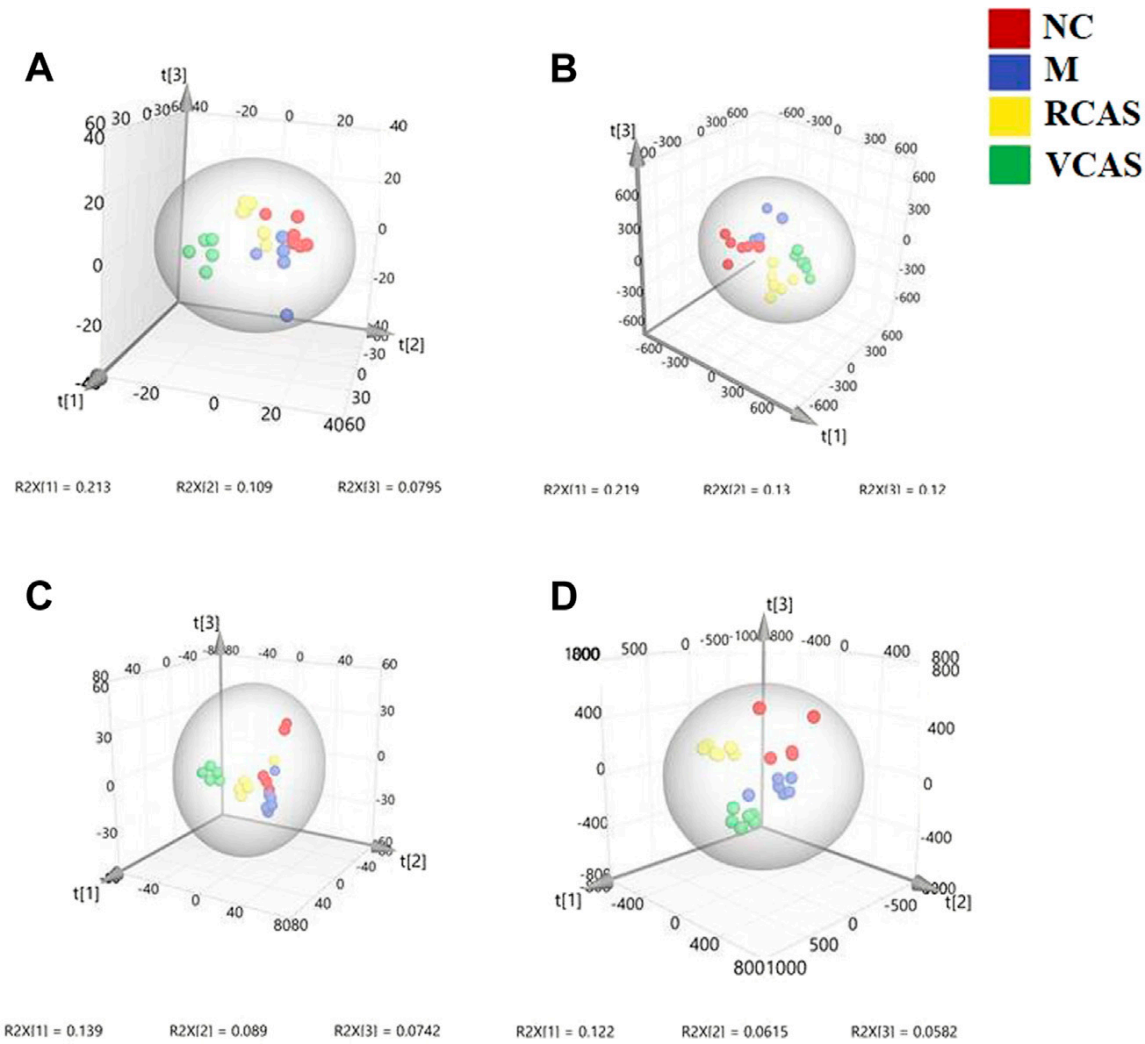
PCA and PLS-DA 3D-score scatter plot of UPLC-Q-TOF/MS data from plasma samples. **(A)** PCA 3D-score scatter plot in the positive ion mode; **(B)** PCA 3D-score scatter plot in the negative ion mode; **(C)** PLS-DA 3D-score scatter plot in the positive ion mode; **(D)** PLS-DA 3D-score scatter plot in negative ion mode.

**FIGURE 4:**
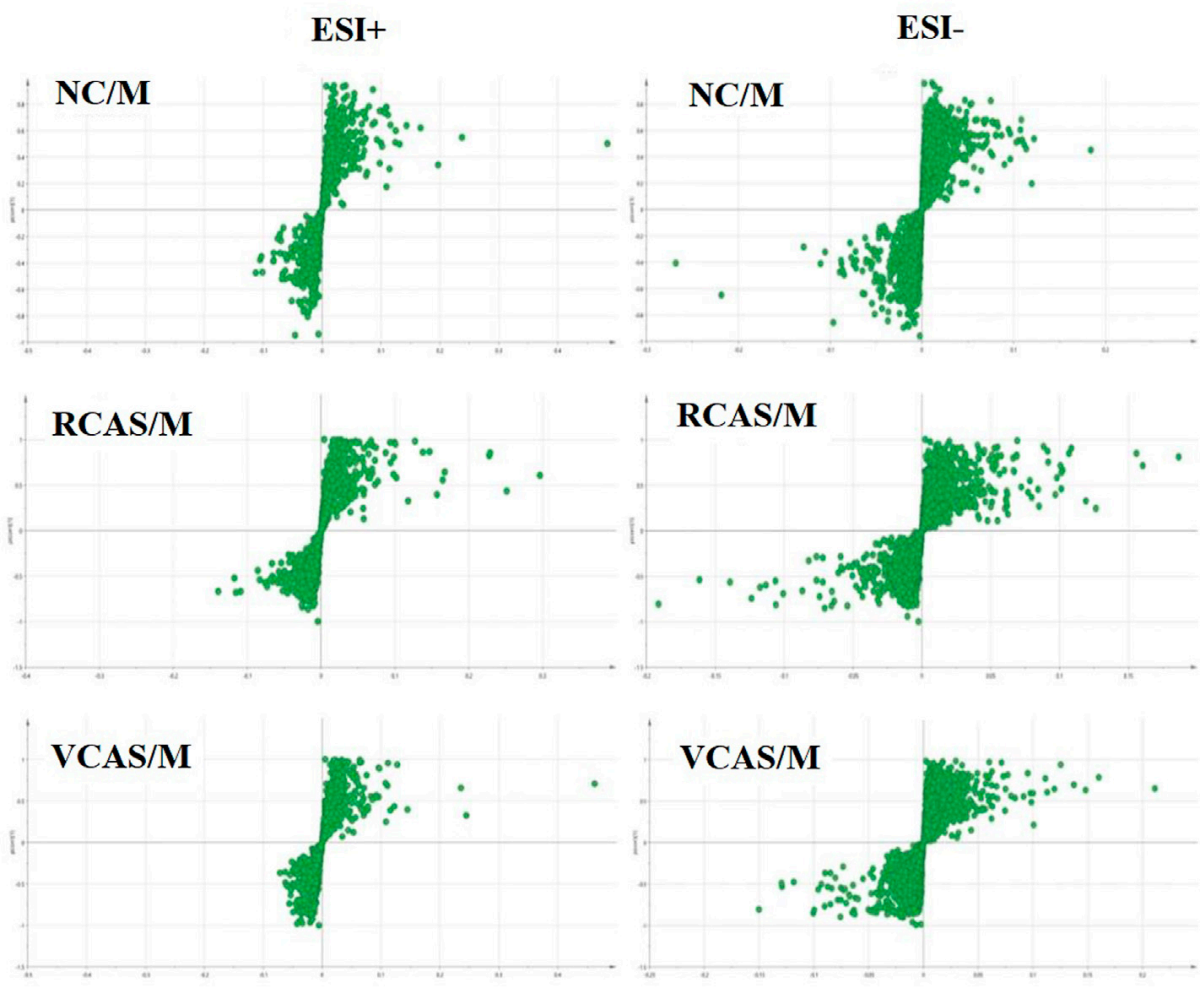
S-plot from plasma samples in the positive and negative ion mode.

In order to deeply excavate the differential endogenous metabolites in plasma samples of each group, the original TIC atlas under positive and negative ion mode was processed by using Marker view 1.2.1 software for dimension reduction, peak matching, and normalization, and further multivariate statistical analysis was carried out to screen the differential markers. The dimension-reduced Excel data (including sample ID, mass-charge ratio/retention time, and ionic strength) were imported into Simca-P 14.1 software for pattern recognition. First, principal component analysis (PCA) was used to summarize the dispersion degree of all samples. The modeling results of 3D-PCA under positive and negative ion modes are shown in Figures 3A,B. Any point in the figure represents a plasma sample. It can be seen from the figure that five groups of plasma samples can be roughly distinguished, but the boundaries are not obvious enough. Therefore, the orthogonal partial least squares method was further used to establish the 3D-PLS-DA model. The results are shown in Figures 3C,D. The grouping of the models modeled by the positive and negative ion modes was obvious, indicating that the endogenous metabolites in plasma samples were significantly different between the groups. Therefore, the OPLS-DA model was established by comparing the NC, the RCAS, and the VCAS groups with the model group. The modeling parameters of each model are shown in Supplementary Table S2. The data show that the modeling results are good. The S-plot graph was further drawn. Each point in the graph represents a “mass-charge ratio/retention time” pair (*m/z*-Rt). The closer the two ends of the “S" curve are, the greater the contribution of ions to the difference between groups. The S-plot plots of plasma samples in each group compared with the model group are shown in Figure 4.

#### 3.3.2 Identification of differential metabolites

In order to obtain the differential metabolites among the plasma samples of each group, the OPLS-DA model needs to be analyzed for the next step of data analysis. In order to improve experimental accuracy, the One-MAP cloud platform was utilized to identify the differential metabolites, the original spectra of all the initial samples after dimension reduction of Excel data (including sample ID, mass/charge ratio/retention time, and ionic strength) and the use of AB SCIEX secondary mass spectra of different substances extracted by the company’s supporting Pekview software were imported into the One-MAP online cloud platform, and differential metabolites were automatically searched and metabolic pathways were analyzed. A variable important for the OPLS-DA model was projected according to each group. Projection, VIP screening of different ions, indicates that the greater the VIP value, the greater the contribution to the grouping. Referring to the literature on metabolomics, this study set a VIP value >1 as the difference between the two groups. In this step, the number of differentiated ions obtained by screening is large, and there is some misjudgment. Therefore, the cloud platform automatically carries out an independent sample *t*-test for the differentiated ions obtained by preliminary screening and rejects the data with *p* > 0.05. Finally, the differential ions with *p* < 0.05 were input into the HMDB database, the structure of compounds was predicted based on the first-order mass spectrometry information, and the predicted exogenous compounds were eliminated to obtain the final selected differentials for structural identification. Each was compared with the standard second-order mass spectrometry data in the KEGG, HMDB, METLIN and other compound databases and relevant literature, and finally comprehensive analysis and identification were obtained. The differential metabolites of group samples are shown in Supplementary Table S3.

Through analysis and identification of differential metabolites and matching with online databases such as HMDB and KEGG, the final 12 endogenous differential metabolites were identified in each group of plasma samples. These metabolites mainly include phenylalanine; phosphatidylcholine, also known as phosphatidylcholine (PC); lysophosphatidylcholine (LysoPC); tryptophan (L-tryptophan); and L-glutamic acid.

#### 3.3.3 Analysis of metabolic pathways

**FIGURE 5:**
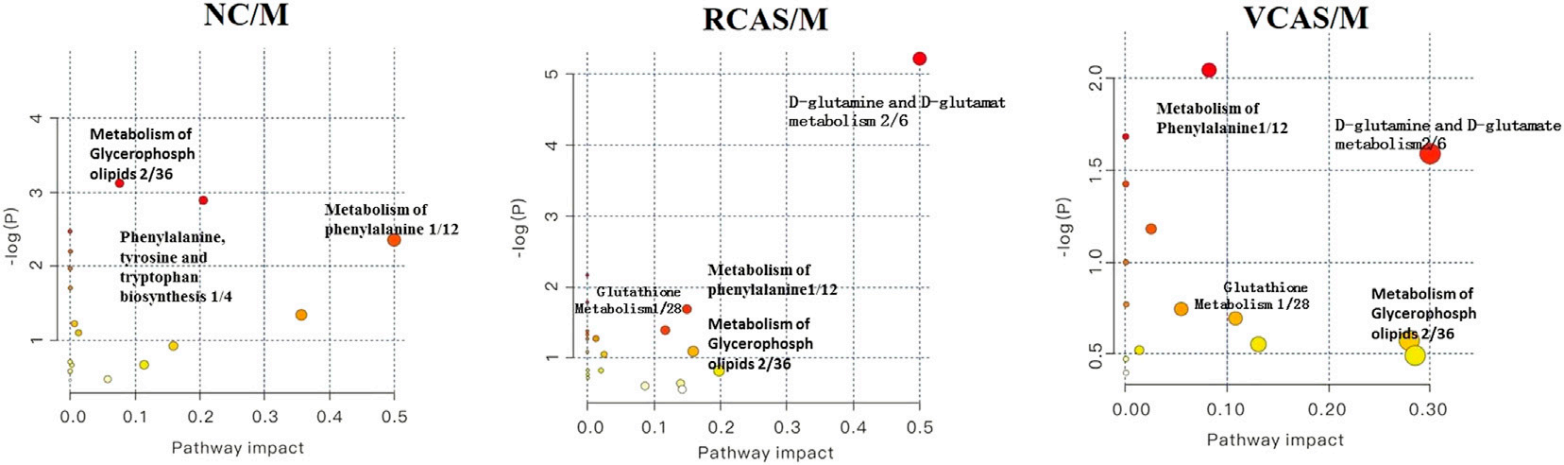
Enrichment results of metabolic pathways between plasma samples.

The endogenous differential metabolites identified earlier were imported into the MetaboAnalyst 4.0 metabolomics online analysis platform (https://www.metaboanalyst.ca/faces/home.xhtml), the *Rattus norvegicus* (rat) pathway library was selected as the metabolic pathway database, the hypergeometric test as pathway enrichment analysis, relative-betweenness pathway topology was analyzed by centrality, and pathway enrichment was performed to screen out metabolic pathways with an impact value greater than 0.10. Pathway results of the blank group, each drug administration group, and the model group are shown in Supplementary Table S4, and the pathway enrichment map is shown in Figure 5. The results showed that the main metabolic pathways involved in the differential metabolites of plasma samples in each group included pyrimidine metabolism, pyruvate metabolism, phenylalanine metabolism, and tyrosine metabolism. The abnormal metabolism of dysmenorrhea rats with blood stasis and the therapeutic effects of warm tulip on blood stasis syndrome were closely related to the aforementioned lipid metabolism pathways and amino acid metabolism pathways.

### 3.4 Urine metabolomics analysis

#### 3.4.1 Multivariate statistical analysis

**FIGURE 6:**
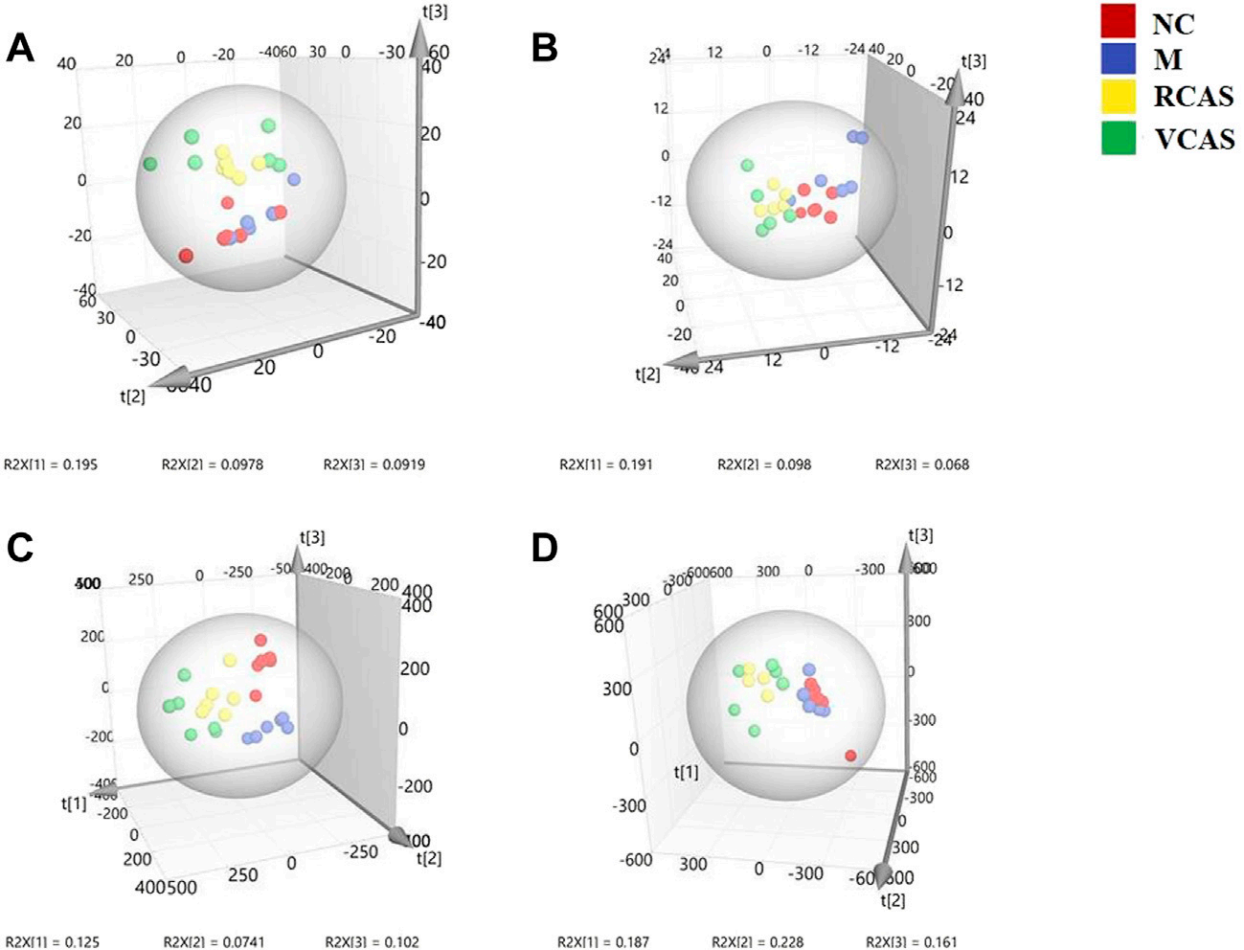
PCA and PLS-DA 3D-score scatter plot of UPLC-Q-TOF/MS data from urine samples. **(A)** PCA 3D-score scatter plot in the positive ion mode; **(B)** PCA 3D-score scatter plot in the negative ion mode; **(C)** PLS-DA 3D-score scatter plot in the positive ion mode; **(D)** PLS-DA 3D-score scatter plot in the negative ion mode.

**FIGURE 7:**
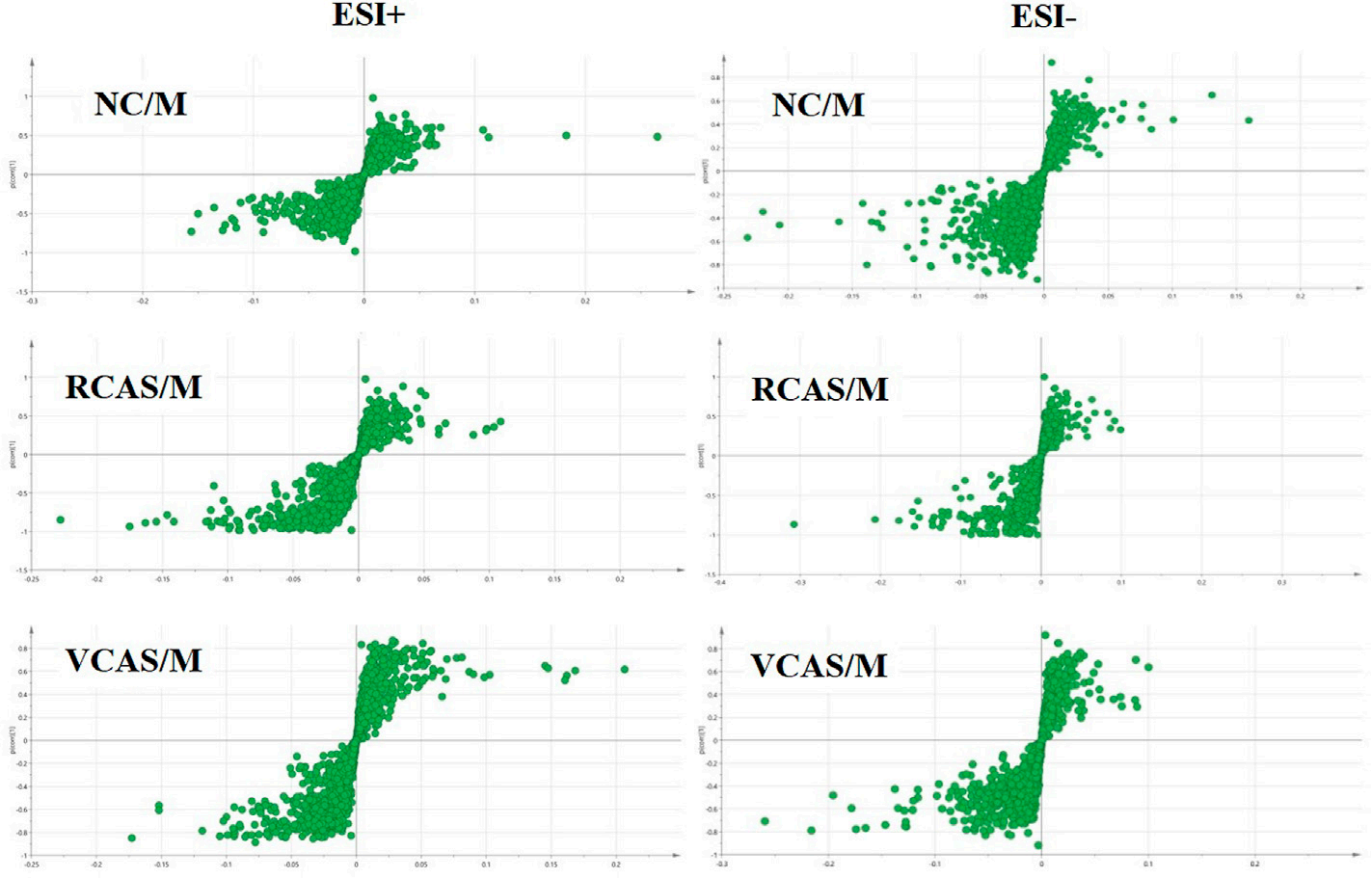
S-plot from urine samples in the positive and negative ion mode.

The modeling results of 3D-PCA under positive and negative ion modes are shown in Figures 6A,B. Any point in the figure represents a urine sample. It can be seen from the figure that five groups of urine samples can be roughly distinguished, but the boundaries are not obvious enough. Therefore, the orthogonal partial least squares method was further used to establish the 3D-PLS-DA model. The results are shown in Figures 6C,D. The grouping of the models modeled by the positive and negative ion modes was obvious, which indicated that the endogenous metabolites in urine samples were significantly different between the groups. Therefore, the OPLS-DA model was established by comparing the NC group, the RCAS group, and the VCAS group with the model group. The modeling parameters of each model are shown in Supplementary Table S5. The data show that the modeling results are good. The S-plot graph was further drawn. Each point in the graph represents a “mass–charge ratio/retention time” pair (*m/z*-Rt). The closer the two ends of the “S” curve are, the greater the contribution of ions to the difference between groups. S-plot plots of urine samples in each group compared with those of the model group are shown in Figure 7.

#### 3.4.2 Identification of different metabolites in urine samples of each group

The same method as mentioned earlier was used to obtain the comprehensive analysis and identification as shown in Supplementary Table S6 for the results of differential metabolites. Through the analysis and identification of differential metabolites and matching with HMDB, KEGG, and other online databases, 19 endogenous differential metabolites were identified in each group of urine samples. These metabolites mainly include phenylalanine; phosphatidylcholine, also known as phosphatidylcholine (PC), lysophosphatidylcholine (lysoPC); and phosphatidic acid (PA) compounds, such as L-tyrosine, steroid hormones, and L-cysteine.

#### 3.4.3 Analysis of metabolic pathways

**FIGURE 8:**
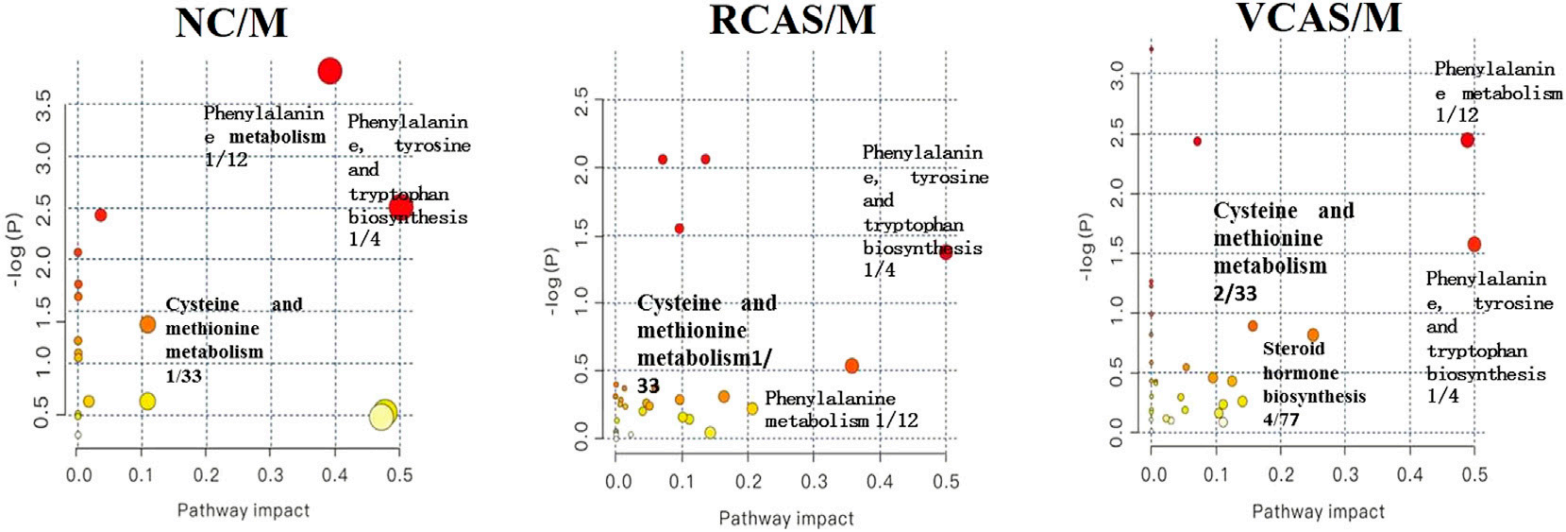
Enrichment results of metabolic pathways between urine samples.

The endogenous differential metabolites identified earlier were input into the MetaboAnalyst 4.0 metabonomics online analysis platform (https://www.metaboanalyst.ca/faces/home.xhtml). *R. norvegicus* (rat) was selected as the metabolic pathway database, and the hypergeometric test as the pathway enrichment analysis centrality analyzed the topological structure of the pathway, enriched the pathway, and screened out the metabolic pathway, with an impact value greater than 0.10. Pathway enrichment maps are shown in Figure 8. The results showed that the main metabolic pathways involved in the different metabolites of urine samples were pyrimidine metabolism, pyruvate metabolism, phenylalanine metabolism, and tyrosine metabolism. The metabolic abnormality of dysmenorrhea rats with blood stasis and the therapeutic effect of three processed products of VCAS on blood stasis syndrome were closely related to the aforementioned lipid metabolic pathway and amino acid metabolic pathway (Supplementary Table S7).

### 3.5 Fecal metabolomics analysis

#### 3.5.1 Multivariate statistical analysis

**FIGURE 9:**
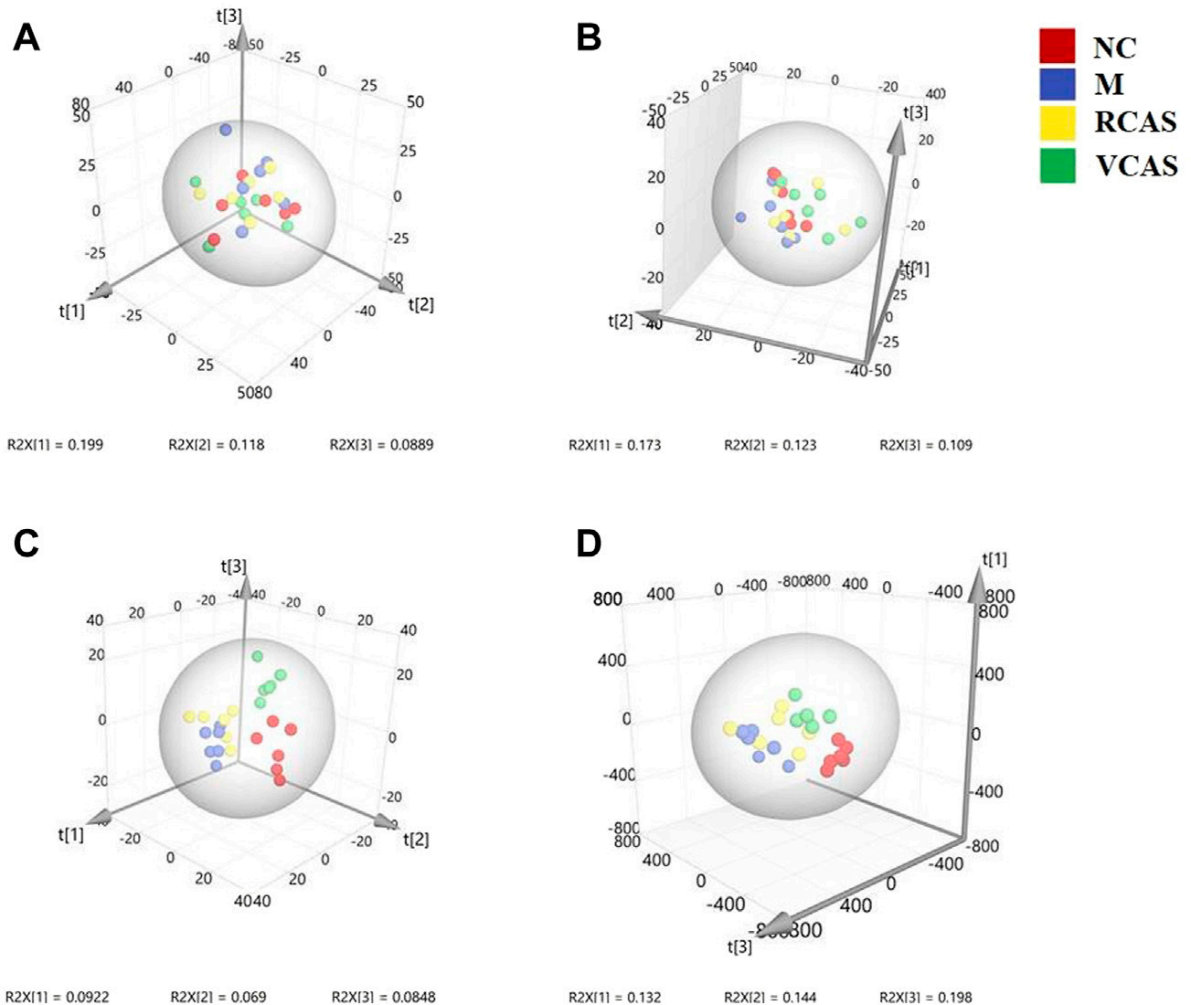
PCA and PLS-DA 3D-score scatter plot of UPLC-Q-TOF/MS data from feces samples. **(A)** PCA 3D-score scatter plot in the positive ion mode; **(B)** PCA 3D-score scatter plot in the negative ion mode; **(C)** PLS-DA 3D-score scatter lot in the positive ion mode; **(D)** PLS-DA 3D-score scatter plot in the negative ion mode.

**FIGURE 10:**
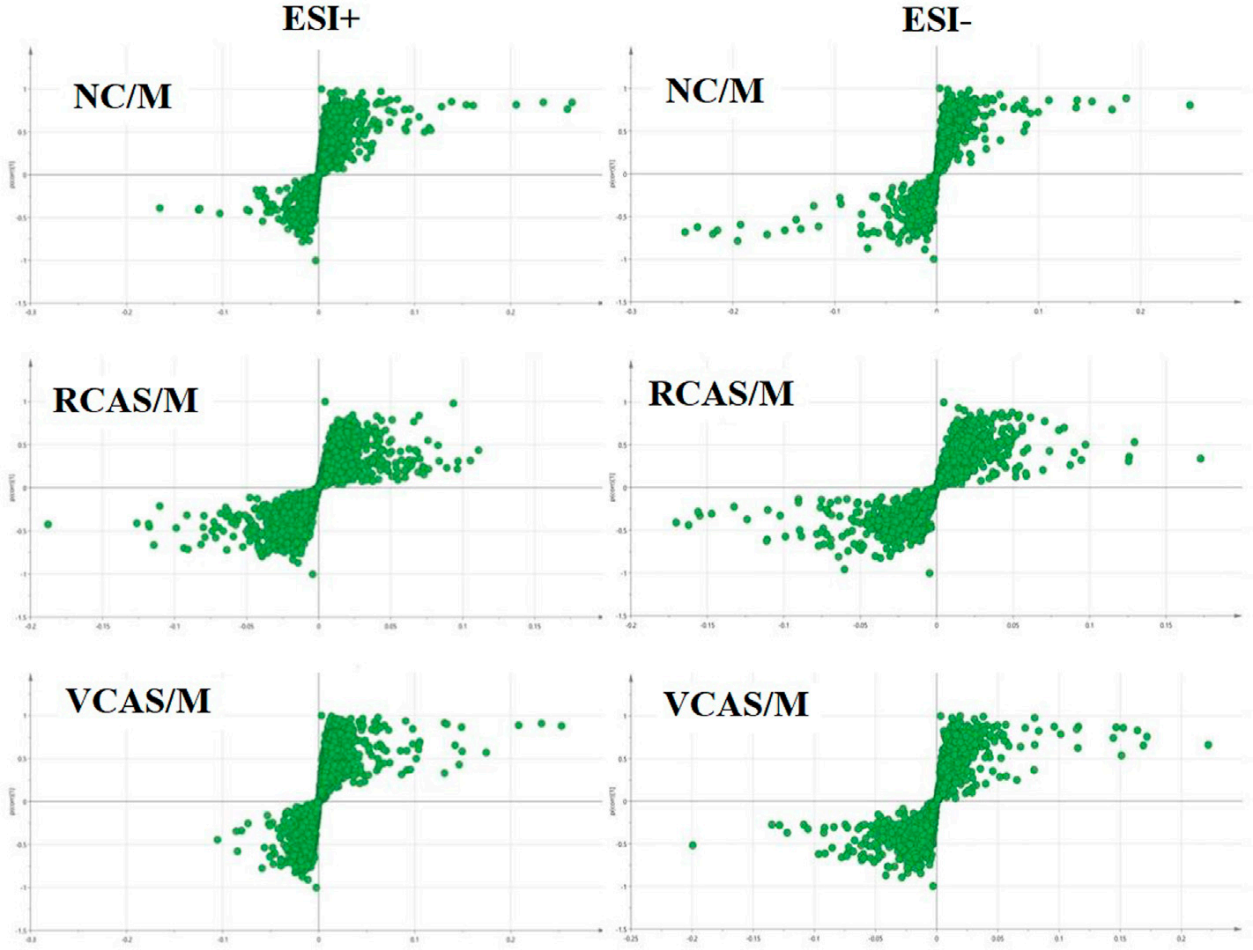
S-plot from feces samples in the positive and negative ion mode.

The modeling results of 3D-PCA under positive and negative ion modes are shown in Figures 9A,B. Any point in the figure represents a fecal sample. It can be seen from the figure that five groups of fecal samples can be roughly distinguished, but the boundaries are not obvious enough. Therefore, the orthogonal partial least squares method was further used to establish the 3D-OPLS-DA model, and the results are shown in Figures 9C,D. The grouping of the models modeled by the positive and negative ion modes was obvious, which indicated that the endogenous metabolites of the fecal samples were different among the groups to some extent. Therefore, the OPLS-DA model was established by comparing the NC group, RCAS group, and VCAS group with the M group. The modeling parameters of each model are shown in Supplementary Table S8. The results show that the modeling results are good. The S-plot graph was further drawn. Each point in the graph represents a “mass-charge ratio/retention time” pair (*m/z*-Rt). The closer the two ends of the “S” curve are, the greater the contribution of ions to the difference between groups. The S-plot of fecal samples in each group compared with that in the model group is shown in Figure 10.

#### 3.5.2 Identification of different metabolites in fecal samples

The results of the differential metabolites are shown in Supplementary Table S9. Through differential metabolite analysis, identification, and matching with online databases such as HMDB and KEGG, 28 endogenous differential metabolites were identified in each group of fecal samples, which mainly included arachidonic acid, also known as leukotriene C4; phenylalanine; phosphatidylcholine, also referred as phosphatidylcholine (PC); sphingomyelin (SM); L-tyrosine; prostaglandin G2; and prostaglandin D2.

#### 3.5.3 Analysis of metabolic pathways of fecal metabolites

**FIGURE 11:**
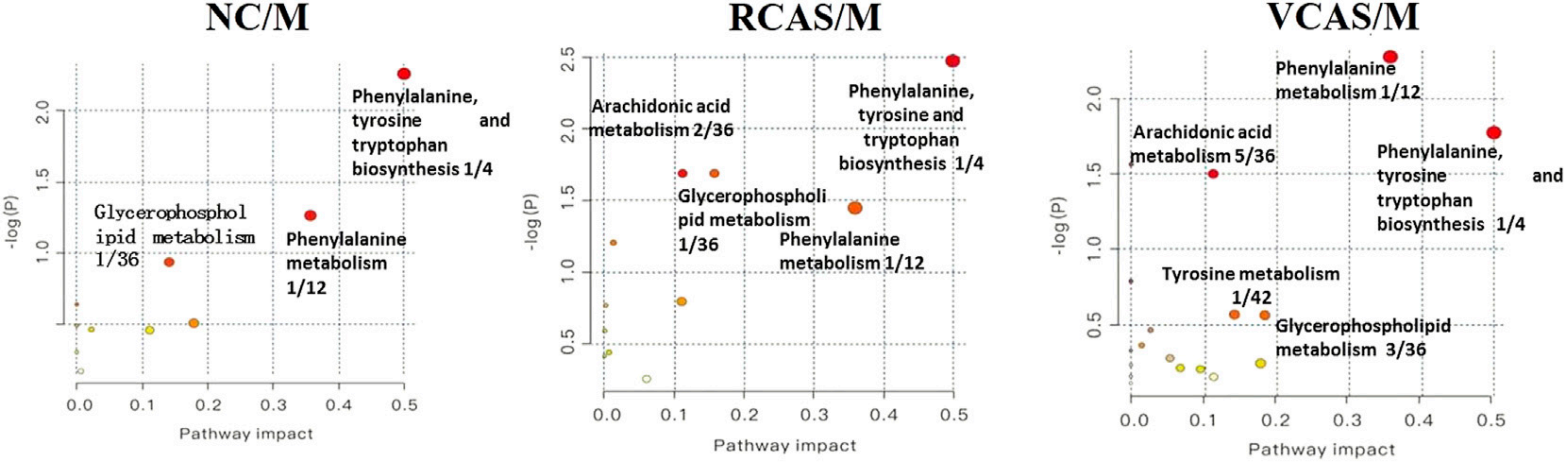
Enrichment results of metabolic pathways between feces samples.

The pathway results of group comparison are shown in Supplementary Table S10, and pathway enrichment maps are shown in Figure 11. The results showed that the metabolic pathways mainly involved in the differential metabolites of fecal samples in each group included glycerophospholipid metabolism, arachidonic acid metabolism, phenylalanine metabolism, and tyrosine metabolism, indicating that the abnormal metabolism of rats with blood stasis syndrome and the therapeutic effect of three processed products of warm tulip on blood stasis syndrome were closely related to the aforementioned lipid metabolism pathways and amino acid metabolism pathways.

## 4 Discussion

The endometrium is an important part of prostaglandin synthesis. It is generally believed that the occurrence of primary dysmenorrhea is mainly related to the increase of prostaglandin synthesis and release in the endometrium during menstruation. Prostaglandin (PG) can be converted into different *p*Gs in the body, such as PGF2 *a* (PGF2 α), PGE2 (PGE2), and TXA2 (thromboxane A2) (Guimarães and Povoa, 2020). PGF2 *a* stimulates uterine contraction, increases uterine tension, and decreases blood flow; on the contrary, PGE2 inhibits uterine contraction, inhibits the spontaneous activity of smooth muscle, and relaxes the uterus (Ruoff and Lema, 2003; Harel, 2004). Therefore, the concentration of PGF2 *a*/PGE2 in the endometrium and blood of dysmenorrhea patients 48 h before menstruation was significantly higher than that of normal people: the more severe the dysmenorrhea, the higher the level of PGF2 *a* (Richter et al., 2006; Dawood and Khan-Dawood, 2007a). In order to reduce the contractility of uterine smooth muscle and achieve the purpose of treating primary dysmenorrhea, it is necessary to inhibit the content of PGF2 *a* and increase the content of PGE2 (Dawood and Khan-Dawood, 2007b; Shi et al., 2011).

Thromboxane B 2 (TXB 2) and 6-keto-prostaglandin F 1 *a* (6-Keto-PGF 1 α) are relatively stable products transformed from two unstable bioactive substances, thromboxane A 2 (TXA 2) and prostacyclin I 2 (PGI 2) (Dawood and Khan-Dawood, 2007b). According to relevant research, TXA 2 is one of the most powerful vasoconstrictors reported *in vivo*, which can enhance platelet activation and aggregation, promote thrombosis, and cause Ca^2+^ influx, thus inducing dysmenorrhea (Das, 2008). Prostacyclin (PGI2) is mainly produced in vascular endothelial cells. By stimulating adenylate cyclooxygenase to increase the level of platelet endogenous camp, it can inhibit platelet aggregation and release induced by ADP, collagen, and AA (Kido et al., 2007), expand blood vessels, prevent platelet aggregation and thrombosis in local blood vessels, relieve dysmenorrhea symptoms, reduce the level of TXB 2 in plasma, increase the content of 6-k-PGF1 α, and reduce the ratio of TXB 2/6-k-PGF1 α so as to inhibit platelet aggregation, expand blood vessels, improve microcirculation, and restore the blood supply of the uterus by activating blood circulation and removing blood stasis so as to relieve dysmenorrhea (Shi et al., 2011).

The expression of TNF-*a* mRNA, protein, and its receptor in human endometrial decidua and trophoblast cells is regulated by estrogen and progesterone (Fluhr et al., 2007). TNF-*a* can inhibit endometrial hyperplasia, induce apoptosis, cause the loss of cadherin catenin actin complex, and promote the injury of endothelial cells. TNF-α, IL-6, and IL-4 can stimulate the AA metabolism of chorionic villi, the latter producing prostaglandins, thromboxane, and leukotrienes. The results showed that the serum levels of IL-6 and IL-10 in dysmenorrhea women were significantly higher than those in non-dysmenorrhea women (Huang et al., 2016; Szebeni et al., 2019). NO is a vasoactive substance, which is the main component of the endothelium relaxing factor. It can dilate blood vessels, and it can reduce thrombosis by inhibiting platelet adhesion, aggregation, and TXB2 release. When the synthesis and release of endogenous NO decrease, the NO-mediated biological information transmission appears abnormal, and the regulation of vasoactive substances is maladjusted, which leads to dysfunction of endothelin function, thus causing pain. As a result, the blood supply of ischemic tissue is gradually restored, and the degree of pain is reduced (Jia et al., 2016).

According to modern medical research, the formation of primary dysmenorrhea may be related to the ischemia–reperfusion injury of the uterine muscle, which causes intracellular Ca^2+^ overload. On the one hand, it will aggravate the contraction of blood vessels and the myometrium and cause the blood supply of the endometrium to be insufficient; on the other hand, it will cause the imbalance of nuclear Ca^2+^ homeostasis, which will lead to depletion of cell energy and damage of the cell membrane, thus causing the production of dysmenorrhea. It can reduce the Ca^2+^ level in uterine smooth muscle cells and relax uterine smooth muscle (Hsia et al., 2008; Thaina et al., 2009; Xiong et al., 2019). β - EP is a class of neuropeptides with morphine-like activity, which is in the endometrial stroma, glandular epithelium, and muscle nerve fibers. It has an endogenous analgesic effect and participates in the regulation of reproductive endocrine. The content of β - EP in peripheral blood of patients with primary dysmenorrhea is significantly higher than that of the NC group (Jia et al., 2006).

VCAS can reduce the ratio of PGF2 *a*/PGE2, TXB2/6-k-PGF1 α, TNF-α, and IL-6 levels, promote the synthesis and release of endogenous NO, reduce the level of Ca^2+^ in uterine smooth muscle cells so as to inhibit platelet aggregation, dilate blood vessels, improve microcirculation, and restore the blood supply of the uterus by activating blood circulation and removing blood stasis so as to relieve dysmenorrhea. The effect of RCAS on PGF2 α, TNF-α, NO, Ca^2+^, and β - EP was not obvious, so the therapeutic effect of RCAS on dysmenorrhea was weaker than that of VCAS.

The results of plasma metabolomics showed that the improvement of dysmenorrhea caused by blood stasis was mainly related to the metabolism of glycerin phospholipid, phenylalanine, glutathione, and tryptophan. The results of urine metabolomics showed that the improvement of dysmenorrhea caused by blood stasis was mainly related to the metabolism of glycerophosphatide, phenylalanine, steroid hormone, cysteine and methionine, arginine, and proline. The results of fecal metabolomics showed that the improvement of dysmenorrhea in blood stasis syndrome was mainly related to the glycerin phospholipid metabolic pathway, phenylalanine metabolic pathway, tyrosine metabolic pathway, and arachidonic acid metabolic pathway.

LysoPCs are important metabolites of lipid metabolism and play an important role in dyslipidemia, diabetes, cancer, atherosclerosis, inflammation, and other diseases (Jackson et al., 2008; Jiang et al., 2013). LysoPC can inhibit transcription activity, tissue factor and NF-κB during coagulation and participate in thrombosis by regulating the expression of tissue factors (Engelmann et al., 1999). Lysophosphatidic acid lysoPA (16:0/0:0) can promote the expression of cyclooxygenase-2 (Guimarães and Povoa, 2020), and cyclooxygenase-2 is the key enzyme for the conversion of arachidonic acid to prostaglandins. Compared with the NC group, the contents of lysoPCs and lysoPA (16:0/0:0) in the M group were significantly increased. After the intervention, the RCAS and VCAS had a downregulation effect on the glycerophosphatidylcholine metabolites, especially the VCAS. The results showed that RCAS and VCAS could reduce the glycerol phospholipid metabolites and inhibit cyclooxygenase-2 by regulating the glycerophosphatidic metabolic pathway. The effect of VCAS on dysmenorrhea was more significant.

Arachidonic acid is a type of polyunsaturated fatty acid, which can mediate the production of inflammatory mediators such as TNF-*a* and IL-1, PGD2, TXA2, and leukotriene, which can induce platelet aggregation, promote blood coagulation, smooth muscle contraction, leukocyte chemotaxis, production of inflammatory cytokines, and immune function (Ma et al., 2013; Burns et al., 2018). The changes of PGG2 and PGD2 metabolites are closely related to the occurrence of primary dysmenorrhea. PGE2 produced and released by the endometrium can inhibit the spontaneous activity of uterine smooth muscle, mediate the cAMP signal molecular transduction pathway, and regulate some neurotransmitters, thus leading to downstream pathway reactions (including reproduction and inflammation) (Iacovides et al., 2015; Guimarães and Povoa, 2020). 12 (s)—HETE is one of the six monohydroperoxy fatty acids produced by non-enzymatic oxidation of arachidonic acid (leukotriene), which produces more stable hydroxyl fatty acid (+/−) 12-HETE through reduction reaction and then participates in host defense reaction and pathophysiological conditions, such as allergy and inflammation. Compared with the endogenous metabolite table, the contents of arachidonic acid, PGE2, PGD2 and 12 (s)—HETE in the M group were significantly higher than those in the NC group. After the intervention of VCAS, the contents of arachidonic acid, PGE2, PGD2 and 12 (s)—HETE in the M group were significantly higher than those in the NC group. Therefore, it is speculated that the pro-inflammatory effect of arachidonic acid may be a contributing factor to the formation of blood stasis syndrome. The arachidonic acid metabolic pathway is one of the important pathways for VCAS to remove blood stasis and relieve pain.

Through UHPLC-Q/TOF-MS and multivariate statistical pattern discrimination, the results showed that normal rats and model rats were discernibly divided into two groups. They exhibited clustering state, which indicated the successful replication of PDM rats from the perspective of metabolomics. In addition, uterine histopathology, the pain factors PGF2 *a*/PGE2 and TXB2 related to dysmenorrhea/6-keto-PGF1 α, IL-6, TNF - α, NO, Ca^2+^, β - EP, and other pharmacodynamic indexes changed before and after the establishment of the model. After RCAS and VCAS intervention, the uterine tissue morphology of dysmenorrhea model rats was improved, and gland hypertrophy and myometrial hyperplasia were reduced as well as neutrophil content. By regulating pain-related factors, the metabolism of glycerophospholipid, glutathione, steroid hormone biosynthesis, cysteine and arginine, and arachidonic acid was regulated, while the spasm of the uterine smooth muscles was relieved.

## 5 Conclusion

This study mainly carried out the metabonomics of plasma, urine, and feces of RCAS and VCAS decoction pieces to remove blood stasis and relieve pain and clarified the metabolic abnormalities of primary dysmenorrhea model rats with *Qi* stagnation and blood stasis, and the therapeutic effects of RCAS and VCAS on primary dysmenorrhea with *Qi* stagnation and blood stasis are closely related to lipid metabolism and amino acid metabolism. It effectively explains the similarities and differences in the clinical efficacy of RCAS and VCAS in removing blood stasis and relieving pain on the whole. RCAS can improve uterine ischemia and treat primary dysmenorrhea, and the effect of VCAS is even better. The related signal pathways and metabolic pathways of RCAS/VCAS in the treatment of primary dysmenorrhea are shown in Supplementary Figure S4. This study provides a basis for further study on the mechanism of VCAS in the treatment of primary dysmenorrhea. Through in-depth analysis of dysmenorrhea-related pain factors, related metabolites, and metabolic pathways, it is found that metabolomics can comprehensively demonstrate the impact of the disease on the whole body, and the selected indicators can holistically reflect the state of the disease and provide a new approach for the study of mechanism, screening, or clinical drug treatment of diseases related to blood stasis syndrome.

## Data Availability

The original contributions presented in the study are included in the article/Supplementary Material; further inquiries can be directed to the corresponding authors.
