# Supplementary material for: Revealing the mechanism of raw and vinegar-processed Curcuma aromatica Salisb. [Zingiberaceae] regulates primary dysmenorrhea in rats via integrated metabolomics
Source: Front Pharmacol. 2022 Sep 13;13:926291. doi: 10.3389/fphar.2022.926291 (PMC9513393; doi:10.3389/fphar.2022.926291)
Supplement: Supplementary file 1 [file DataSheet1.docx]

**1 Analysis of chemical constituents of RCAS and VCAS**

*1.1 Analysis conditions of GC-MS*

Chromatographic conditions: HP-INNOWax column (30 m × 0.25 mm, 0.25 μ m); carrier gas is helium, flow rate is 1.0 ml / min, injection port temperature is 240 ℃, split ratio is 3:1; temperature programming: starting temperature is 80 ℃, holding for 1 min, then heating to 150 ℃ at 15 ℃ / min for 1 min, and then heating to 250 ℃ at 6 ℃ / min for 5 min.

Mass spectrometry conditions: EI ion source, electron energy of 70 EV, ion source temperature of 230 ℃, four stage rod temperature of 150 ℃, full scan mode, scanning range of 29 ~ 350 amu.

*1.2 Fingerprint analysis of RCAS and VCAS*

GC-MS method was used to analyze the fingerprints of RCAS and VCAS (Figure S1). The common mode was generated by similarity evaluation system of chromatographic fingerprint of traditional Chinese medicine (2012.130723), and the similarity of RCAS and VCAS decoction pieces were calculated, respectively. The similarity of ten batches of raw and vinegar-processed *Curcuma aromatica Salisb*. [Zingiberaceae] decoction pieces were greater than 0.90, indicating good consistency of samples. The original data of GC-MS fingerprint of RCAS and VCAS were modeled to obtain PLS-DA score chart (Figure S2). The pattern recognition results showed that RCAS and VCAS could be obviously divided into two categories.


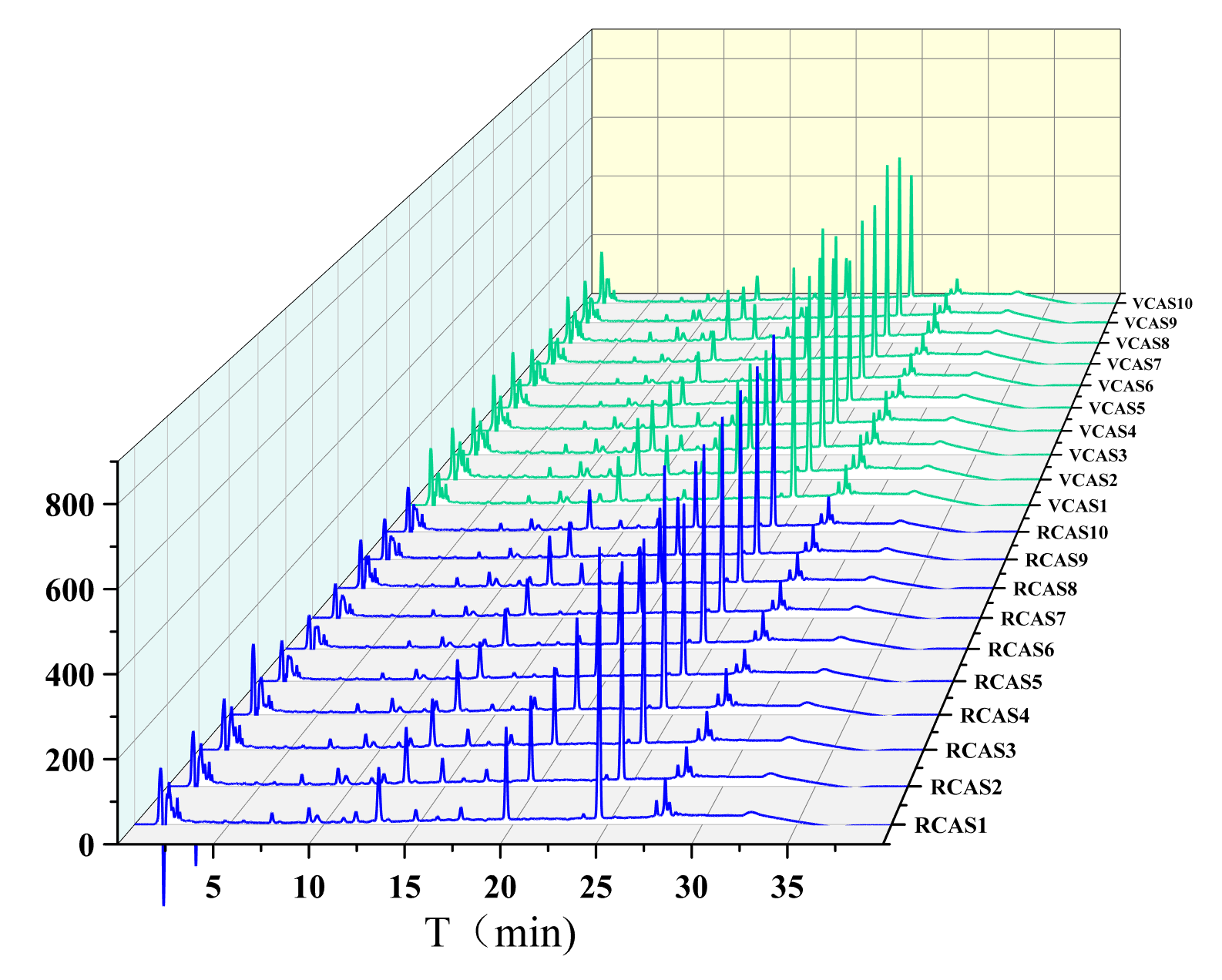


**Figure S1** GC-MS fingerprint overlay of RCAS and VCAS


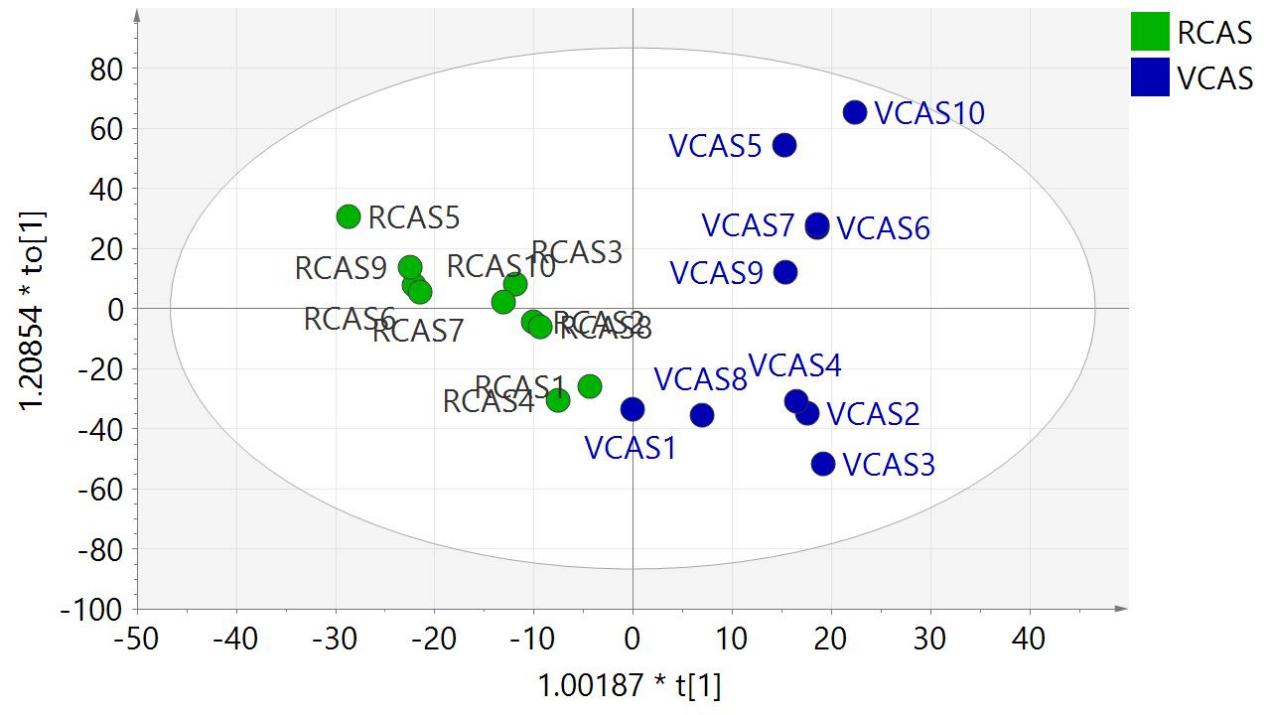


**Figure S2** PLS-DA score chart of RCAS and VCAS

*1.3 Analysis of volatile components in RCAS and VCAS*

A total of 17 compounds were identified by GC-MS (Figure S3), and their relative contents were significantly changed. Among them, curdione, curcumenone, curcumenol, β - elemene, germacrone, etc. were common components in RCAS and VCAS. β - selene was the unique compound of RCAS, while cypera-2 was the specific compound of VCAS. The contents of curdione, epicurzerenone, germacrone, curcumene and β - elemene were relatively high, and the contents of curdione, epicurzerenone and curcumene increased after vinegar processing, while the contents of β - elemene and germacrone decreased after vinegar treatment.

The active components in RCAS and VCAS are mainly sesquiterpenoids. Literature studies have shown that many sesquiterpenoids have heat sensitive properties. Among them, furadiene and gemmatone have 1,4 diene structure in the chemical structure skeleton, which can pass through [3-3] when heated σ The translocation reaction rearranges and gemmatone is converted to β- Elemenone, furadiene is converted to furanoelemene [1]. (4S, 5S) - (+) -Germacrone-4,5-epoxide can be converted to Curcumenone by transcyclization reaction under heating conditions [2].

[1] Baldovini, N., Tomi, F. and Casanova, J. Identification and quantitative determination of

furanodiene, a heat-sensitive compound, in essential oil by 13C-NMR[J]. Phytochem Anal, 2001,

12(1): 58-63.

[2] Kuroyanagi, M., Shirota, O., Sekita, S., Nakane, T. Transannular cyclization of (4S,5S)-

germacrone-4,5-epoxide into guaiane and secoguaianetype sesquiterpenes[J]. Natural Product

Communication, 2012, 7: 441–446.


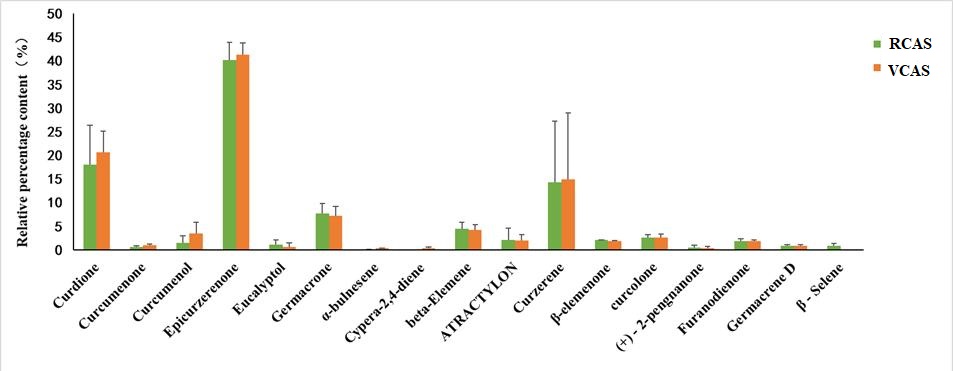


**Figure S3** Relative content analysis of 17 components in RCAS and VCAS

**Table S1** The degree of uterine lesions in each group

| Group | n | Degree of uterine lesions | | | | | Mean | SD | P value |  |
| --- | --- | --- | --- | --- | --- | --- | --- | --- | --- | --- |
|  |  | - | + | ++ | +++ | ++++ |  |  |  |  |
| NC | 10 | 10 | 0 | 0 | 0 | 0 | 0.00 | 0.00 | / |  |
| M | 10 | 0 | 0 | 2 | 2 | 6 | 3.40 | 0.84 | 0 | ## |
| TJB | 10 | 0 | 4 | 3 | 2 | 1 | 2.00 | 1.05 | 0.004 | ** |
| RCAS | 10 | 0 | 1 | 3 | 2 | 4 | 2.90 | 1.10 | 0.269 |  |
| VCAS | 10 | 0 | 3 | 4 | 2 | 1 | 2.10 | 0.99 | 0.006 | ** |

**Table S2** Parameters of PCA and OPLS-DA models of Plasma samples of each group

| **No.** | **Model** | **ESI+** | | |  | **ESI-** | | |
| --- | --- | --- | --- | --- | --- | --- | --- | --- |
|  |  | R^2^X | R^2^Y | Q^2^ |  | R^2^X | R^2^Y | Q^2^ |
|  | PCA | 0.439 |  | 0.00903 |  | 0.302 |  | 0.0823 |
|  | PLS-DA | 0.29 | 0.359 | 0.0761 |  | 0.242 | 0.641 | 0.234 |
| NC/M | OPLS-DA | 0.122 | 0.875 | -0.242 |  | 0.423 | 0.92 | 0.312 |
| RCAS/M | OPLS-DA | 0.798 | 1 | 0.924 |  | 0.769 | 1 | 0.649 |
| VCAS/M | OPLS-DA | 0.881 | 1 | 0.852 |  | 0.43 | 0.972 | 0.657 |

**Table S3** 9 differential metabolites identified in Plasma samples

| No. | HMDB ID | Average Mass | Adduct ion | ppm | Formula | Endogenous Metabolite | T_a_ | T_b_ | T_c_ |
| --- | --- | --- | --- | --- | --- | --- | --- | --- | --- |
| M_1_ | HMDB0000929 | 204.2252 |  |  | C_11_H_12_N_2_O_2_ | L-Tryptophan | - | - | ↓ |
| M_2_ | HMDB0000159 | 165.1891 |  |  | C_9_H_11_NO_2_ | L-Phenylalanine | ↑ | ↓ | ↓ |
| M_3_ | HMDB0008189 | 828.1086 |  |  | C_48_H_78_NO_8_P | PC(18:3(6Z,9Z,12Z)/-22:6(4Z,7Z,10Z,13Z,16Z,19Z)) | ↓ | ↑ | ↑ |
| M_4_ | HMDB0003134 | 372.483 |  |  | C_16_H_28_N_4_O_4_S | Biocytin | ↓ | ↑ | ↑ |
| M_5_ | HMDB0000641 | 146.1445 |  |  | C_5_H_10_N_2_O_3_ | 1. Glutamic acid | - | ↑ | ↑ |
| M_6_ | HMDB0000641 | 146.1445 |  |  | C_5_H_10_N_2_O_3_ | D-Glutamine | ↓ | ↑ | ↑ |
| M_7_ | HMDB0000821 | 193.1992 |  |  | C_10_H_11_NO_3_ | Phenylacetylglycine | - | ↓ | - |
| M_8_ | HMDB0007972 | 760.0761 |  |  | C_42_H_82_NO_8_P | PC(16:0/18:1(9Z)) | - | ↑ | ↑ |
| M_9_ | HMDB0008090 | 832.1403 |  |  | C_48_H_82_NO_8_P | PC(18:1(11Z)/-22:6(4Z,7Z,10Z,13Z,16Z,19Z)) | - | ↑ | ↑ |

T_a_: NC/M, T_b_: RCAS/M, T_c_: VCAS/M.

**Table S4** Metabolic Pathways of Plasma Samples

| **Metabolic pathways** | **Total** | **Hits** | **Raw p** | **-LOG (p)** | **Holm adjust** | **FDR** | **Impact** |
| --- | --- | --- | --- | --- | --- | --- | --- |
| Phenylalanine, tyrosine and tryptophan biosynthesis tryptophan biosynthesis | 4 | 1 | 0.107 | 2.24 | 1 | 1 | 0.5 |
| D-Glutamine and D-glutamate metabolism | 6 | 2 | 0.00546 | 5.21 | 0.458 | 0.458 | 0.5 |
| Glutathione metabolism | 28 | 1 |  |  | 1 | 1 | 0.12 |
| Phenylalanine metabolism | 12 | 1 | 0.258 | 1.35 | 1 | 1 | 0.36 |
| Glycerol phospholipid metabolism | 36 | 2 | 0.176 | 1.74 | 1 | 1 | 0.23 |
| Alanine, aspartate and glutamate metabolism | 28 | 1 | 0.433 | 0.837 | 1 | 1 | 0.2 |
| Selenocompound metabolism | 20 | 1 | 0.393 | 0.933 | 1 | 1 | 0.16 |
| Biotin metabolism | 10 | 1 | 0.247 | 1.4 | 1 | 1 | 0.15 |
| Tryptophan metabolism | 41 | 1 | 0.691 | 0.37 | 1 | 1 | 0.14 |
| phosphoinositide metabolism | 30 | 1 | 0.478 | 0.739 | 1 | 1 | 0.13 |
| Arginine biosynthesis | 14 | 1 | 0.246 | 1.4 | 1 | 1 | 0.12 |
| galactose metabolism | 27 | 1 | 0.411 | 0.89 | 1 | 1 | 0.11 |
| Alanine, aspartate and glutamate metabolism | 28 | 1 | 0.504 | 0.685 | 1 | 1 | 0.11 |

**Table S5** Parameters of PCA and OPLS-DA models of Urine samples of each group

| No. | Model | ESI+ | | |  | ESI- | | |
| --- | --- | --- | --- | --- | --- | --- | --- | --- |
|  |  | R^2^X | R^2^Y | Q^2^ |  | R^2^X | R^2^Y | Q^2^ |
|  | PCA | 0.634 |  | 0.0393 |  | 0.567 |  | 0.0904 |
|  | PLS-DA | 0.31 | 0.317 | 0.139 |  | 0.643 | 0.444 | 0.82 |
| NC/M | OPLS-DA | 0.419 | 0.962 | 0.754 |  | 0.817 | 0.858 | 0.417 |
| RCAS/M | OPLS-DA | 0.738 | 0.966 | 0.436 |  | 0.937 | 0.999 | 0.901 |
| VCAS/M | OPLS-DA | 0.642 | 0.689 | 0.492 |  | 0.763 | 0.845 | 0.749 |

**Table S6** 19 differential metabolites identified in fecal samples

| **No.** | **HMDB ID** | Average Mass | Adduct ion | ppm | **Formula** | **Endogenous Metabolite** | **T_a_** | **T_b_** | **T_c_** |
| --- | --- | --- | --- | --- | --- | --- | --- | --- | --- |
| M_1_ | HMDB0000159 | 165.1891 |  |  | C_9_H_11_NO_2_ | L-Phenylalanine | ↑ | ↓ | ↓ |
| M_2_ | HMDB0000821 | 193.1992 |  |  | C_10_H_11_NO_3_ | Phenylacetylglycine | - | ↓ | - |
| M_3_ | HMDB0007972 | 760.0761 |  |  | C_42_H_82_NO_8_P | PC(16:0/18:1(9Z)) | - | ↑ | ↑ |
| M_4_ | HMDB0008090 | 832.1403 |  |  | C_48_H_82_NO_8_P | PC(18:1(11Z)/-22:6(4Z,7Z,10Z,13Z,16Z,19Z)) | - | ↑ | ↑ |
| M_5_ | HMDB0000574 | 121.158 |  |  | C_3_H_7_NO_2_S | L-Cysteine | ↓ | - | ↑ |
| M_6_ | HMDB0006834 | 322.378 |  |  | C_12_H_22_N_2_O_6_S | D-pantothenate-cysteine | - | ↑ | ↑ |
| M_7_ | HMDB0001202 | 307.1971 |  |  | C_9_H_14_N_3_O_7_P | dCMP | - | - | ↓ |
| M_8_ | HMDB0000218 | 368.1908 |  |  | C_10_H_13_N_2_O_11_P | Orotidylic acid | - | ↑ | ↑ |
| M_9_ | HMDB0000012 | 228.202 |  |  | C_9_H_12_N_2_O_5_ | Deoxyuridine | - | ↑ | ↑ |
| M_10_ | HMDB0000099 | 222.262 |  |  | C_7_H_14_N_2_O_4_S | L-cystathionine | - | ↑ | ↑ |
| M_11_ | HMDB0000177 | 155.1546 |  |  | C_6_H_9_N_3_O_2_ | L-Histidine | - | - | ↑ |
| M_12_ | HMDB0000214 | 132.161 |  |  | C_5_H_12_N_2_O_2_ | Ornithine | - | ↑ | ↑ |
| M_13_ | HMDB0000016 | 330.4611 |  |  | C_21_H_30_O_3_ | Deoxycorticosterone | - | - | ↑ |
| M_14_ | HMDB0004029 | 344.4446 |  |  | C_21_H_28_O_4_ | 11-Dehydrocorticosterone | - | - | ↑ |
| M_15_ | HMDB0001032 | 368.488 |  |  | C_19_H_28_O_5_S | Dehydroepiandrosterone sulfate | - | ↑ | ↑ |
| M_16_ | HMDB0000053 | 286.4085 |  |  | C_19_H_26_O_2_ | Androstenedione | - | ↓ | ↓ |
| M_17_ | HMDB0007853 | 410.4825 |  |  | C_19_H_39_O_7_P | LysoPA(16:0/0:0) | - | ↑ | ↑ |
| M_18_ | HMDB0007855 | 436.526 |  |  | C_21_H_41_O_7_P | LysoPA(18:1(9Z)/0:0) | ↓ | ↑ | ↑ |
| M_19_ | HMDB0000243 | 88.0621 |  |  | C_3_H_4_O_3_ | pyruvic acid | - | ↓ | ↓ |

**Table S7** Metabolic Pathways of Urine Samples

| **Metabolic pathways** | **Total** | **Hits** | **Raw p** | **-log(p)** | **Holm adjust** | **FDR** | **Impact** |
| --- | --- | --- | --- | --- | --- | --- | --- |
| Phenylalanine, tyrosine and tryptophan biosynthesis | 4 | 1 | 0.301 | 1.2 | 1 | 1 | 0.5 |
| Phenylalanine metabolism | 12 | 1 | 0.234 | 0.56 | 1 | 1 | 0.36 |
| Pyrimidine metabolism | 39 | 3 | 0.663 | 0.412 | 1 | 1 | 0.16 |
| Histidine metabolism | 16 | 1 | 0.762 | 0.271 | 1 | 1 | 0.22 |
| Cysteine and methionine metabolism | 33 | 2 | 0.789 | 0.237 | 1 | 1 | 0.27 |
| Steroid hormone biosynthesis | 77 | 5 | 0.805 | 0.217 | 1 | 1 | 0.17 |
| Arginine and proline metabolism | 38 | 2 | 0.851 | 0.161 | 1 | 1 | 0.19 |
| Tyrosine metabolism | 42 | 1 | 0.978 | 0.0225 | 1 | 1 | 0.14 |
| Glycerophospholipid metabolism | 36 | 1 | 0.27 | 1.31 | 1 | 1 | 0.14 |
| Pyruvate metabolism | 22 | 1 | 0.15 | 1.9 | 1 | 1 | 0.21 |
| Glycolysis / Gluconeogenesis | 26 | 1 | 0.175 | 1.75 | 1 | 1 | 0.1 |

**Table S8** Parameters of PCA and OPLS-DA models of fecal samples of each group

| No. | Model | ESI+ | | |  | ESI- | | |
| --- | --- | --- | --- | --- | --- | --- | --- | --- |
|  |  | R^2^X | R^2^Y | Q^2^ |  | R^2^X | R^2^Y | Q^2^ |
|  | PCA | 0.627 |  | 0.224 |  | 0.506 |  | 0.161 |
|  | PLS-DA | 0.885 | 1 | 0.947 |  | 0.792 | 0.999 | 0.834 |
| NC/M | OPLS-DA | 0.895 | 1 | 0.89 |  | 0.9 | 1 | 0.946 |
| RCAS/M | OPLS-DA | 0.851 | 1 | 0.411 |  | 1 | 1 | 0.619 |
| VCAS/M | OPLS-DA | 0.824 | 1 | 0.728 |  | 0.671 | 0.994 | 0.839 |

**Table S9** 18 differential metabolites identified in fecal samples

|  | **HMDBID** | Average Mass | Adduct ion | ppm | **Formula** | **Endogenous Metabolite** | **T_a_** | **T_b_** | **T_c_** |
| --- | --- | --- | --- | --- | --- | --- | --- | --- | --- |
| M_1_ | HMDB0008189 | 828.1086 |  |  | C_48_H_78_NO_8_P | PC(18:3(6Z,9Z,12Z)/-22:6(4Z,7Z,10Z,13Z,16Z,19Z) | ↓ | ↑ | ↑ |
| M_2_ | HMDB0008156 | 830.1245 |  |  | C4_8_H_80_NO_8_P | PC(18:2(9Z,12Z)/-22:6(4Z,7Z,10Z,13Z,16Z,19Z)) | - | - | ↑ |
| M_3_ | HMDB0001043 | 304.4669 |  |  | C_20_H_32_O_2_ | Arachidonic acid | ↓ | - | ↓ |
| M_4_ | HMDB0003235 | 368.4645 |  |  | C_20_H_32_O_6_ | Prostaglandin G2 | ↓ | - | ↓ |
| M_5_ | HMDB0001403 | 352.4651 |  |  | C_20_H_32_O_5_ | Prostaglandin D2 | ↓ | ↓ | ↓ |
| M_6_ | HMDB0001198 | 625.774 |  |  | C_30_H_47_N_3_O_9_S | Leukotriene C4 | - | ↑ | ↑ |
| M_7_ | HMDB0000158 | 181.1885 |  |  | C_9_H_11_NO_3_ | L-Tyrosine | - | - | ↓ |
| M_8_ | HMDB0000641 | 146.1445 |  |  | C_5_H_10_N_2_O_3_ | L-Glutamine | - | - | ↓ |
| M_9_ | HMDB0007856 | 434.51 |  |  | C_21_H_39_O_7_P | LysoPA(18:2(9Z,12Z)/0:0) | - | - | ↑ |
| M_10_ | HMDB0007854 | 438.542 |  |  | C_21_H_43_O_7_P | LysoPA(18:0/0:0) | - | - | ↑ |
| M_11_ | HMDB0004259 | 264.2771 |  |  | C_13_H_16_N_2_O_4_ | Acetyl-N-formyl-5-methoxykynurenamine | - | ↑ | - |
| M_12_ | HMDB0000239 | 169.1778 |  |  | C_8_H_11_NO_3_ | Pyridoxine | - | - | ↑ |
| M_13_ | HMDB0004243 | 336.4657 |  |  | C_20_H_32_O_4_ | 12(S)-HPETE | ↓ | ↑ | ↑ |
| M_14_ | HMDB0007853 | 410.4825 |  |  | C_19_H_39_O_7_P | LysoPA(16:0/0:0) | ↑ | - | ↓ |
| M_15_ | HMDB0000159 | 165.1891 |  |  | C_9_H_11_NO_2_ | L-Phenylalanine | ↑ | ↓ | ↓ |
| M_16_ | HMDB0010316 | 327.2867 |  |  | C_14_H_17_NO_8_ | Acetaminophen glucuronide | - | - | ↑ |
| M_17_ | HMDB0000099 | 222.262 |  |  | C_7_H_14_N_2_O_4_S | L-Cystathionine | - | ↑ | - |
| M_18_ | HMDB0000209 | 136.1479 |  |  | C_8_H_8_O_2_ | Phenylacetic acid | - | - | ↓ |

**Table S10** Metabolic Pathways of Fecal Samples

| **Metabolic pathways** | **Total** | **Hits** | **Raw p** | **-log(p)** | **Holm adjust** | **FDR** | **Impact** |
| --- | --- | --- | --- | --- | --- | --- | --- |
| Phenylalanine, tyrosine and tryptophan biosynthesis | 4 | 1 | 0.227 | 1.48 | 1 | 1 | 0.5 |
| Arachidonic acid metabolism | 36 | 5 | 0.0684 | 2.68 | 1 | 1 | 0.46 |
| Phenylalanine metabolism | 12 | 1 | 0.234 | 1.45 | 1 | 1 | 0.36 |
| Glycerophospholipid metabolism | 36 | 3 | 0.391 | 0.938 | 1 | 1 | 0.34 |
| Tyrosine metabolism | 42 | 3 | 0.493 | 0.708 | 1 | 1 | 0.18 |
| Cysteine and methionine metabolism | 33 | 1 | 0.79 | 0.235 | 1 | 1 | 0.18 |
| Pentose and glucuronate inter-conversions | 18 | 1 | 0.571 | 0.56 | 1 | 1 | 0.14 |
| Alanine, aspartate and glutamate metabolism | 28 | 1 | 0.838 | 0.177 | 1 | 1 | 0.11 |


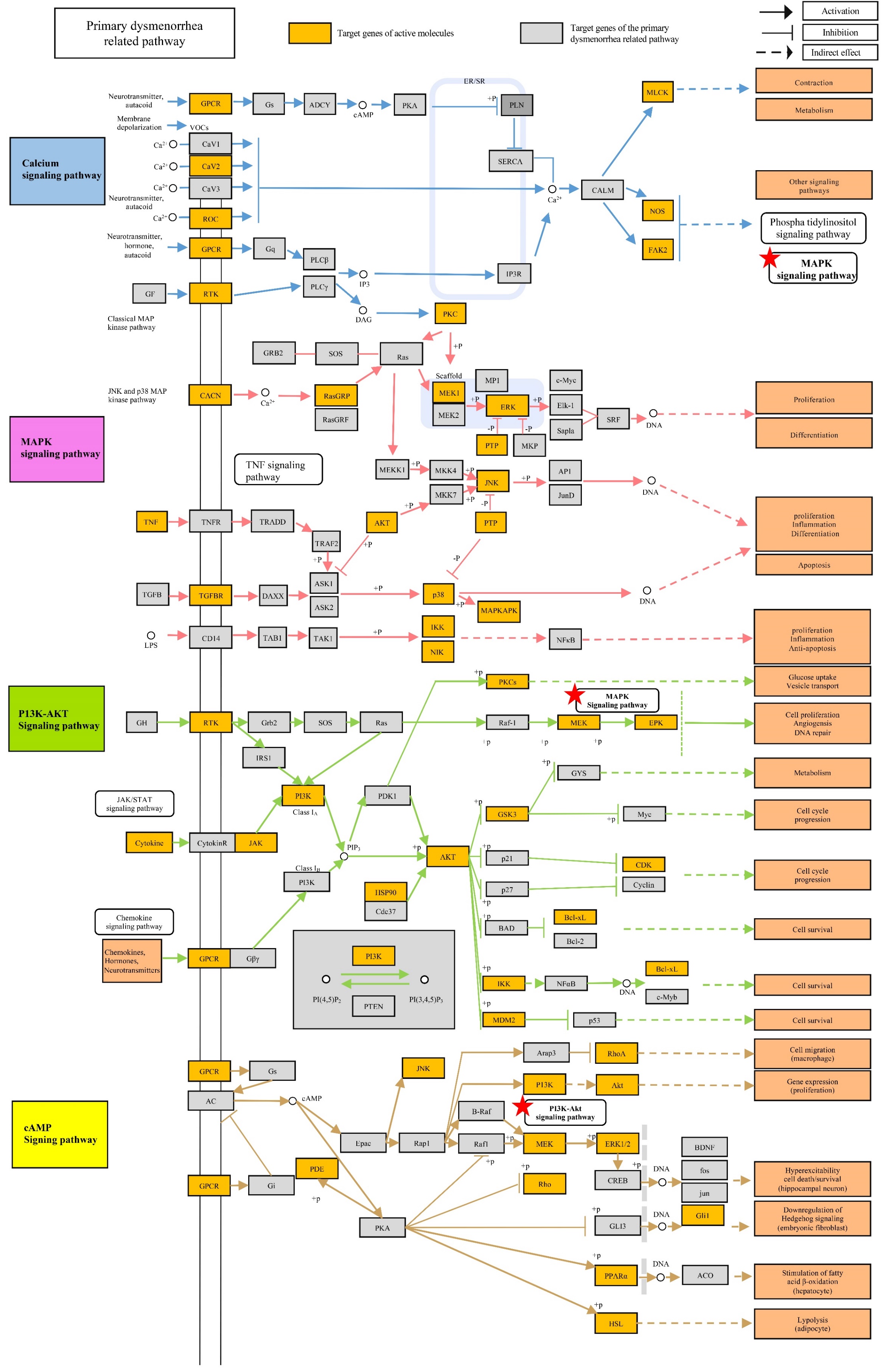


**Figure S4** Related signal pathways and metabolic pathways of RCAS/VCAS in the treatment of primary dysmenorrhea.
